# An *O*-GlcNAc transferase pathogenic variant linked to intellectual disability affects pluripotent stem cell self-renewal

**DOI:** 10.1242/dmm.049132

**Published:** 2023-06-19

**Authors:** Michaela Omelková, Christina Dühring Fenger, Marta Murray, Trine Bjørg Hammer, Veronica M. Pravata, Sergio Galan Bartual, Ignacy Czajewski, Allan Bayat, Andrew T. Ferenbach, Marios P. Stavridis, Daan M. F. van Aalten

**Affiliations:** ^1^ Division of Molecular, Cell and Developmental Biology, School of Life Sciences, University of Dundee, Dundee DD1 5EH, UK; ^2^ Department of Epilepsy Genetics, Filadelfia Danish Epilepsy Centre, Dianalund 4293, Denmark; ^3^ Amplexa Genetics A/S, Odense 5000, Denmark; ^4^ Institute of Molecular Precision Medicine, Xiangya Hospital, Central South University, Changsha 410008, China; ^5^ Department of Molecular Biology and Genetics, Aarhus University, Aarhus 8000, Denmark

**Keywords:** OGT, *O*-GlcNAc, Congenital disorders of glycosylation, Intellectual disability, Stem cells, Self-renewal

## Abstract

*O*-linked β-*N*-acetylglucosamine (*O*-GlcNAc) transferase (OGT) is an essential enzyme that modifies proteins with *O*-GlcNAc. Inborn *OGT* genetic variants were recently shown to mediate a novel type of congenital disorder of glycosylation (OGT-CDG), which is characterised by X-linked intellectual disability (XLID) and developmental delay. Here, we report an OGT^C921Y^ variant that co-segregates with XLID and epileptic seizures, and results in loss of catalytic activity. Colonies formed by mouse embryonic stem cells carrying OGT^C921Y^ showed decreased levels of protein *O*-GlcNAcylation accompanied by decreased levels of Oct4 (encoded by *Pou5f1*), Sox2 and extracellular alkaline phosphatase (ALP), implying reduced self-renewal capacity. These data establish a link between OGT-CDG and embryonic stem cell self-renewal, providing a foundation for examining the developmental aetiology of this syndrome.

## Teaser

**Summary:** The C921Y *O*-GlcNAc transferase variant found in patients with intellectual disability leads to a defect in pluripotent stem cell self-renewal and decreased levels of pluripotent stem cell markers.

## INTRODUCTION

Intellectual disability (ID) affects up to 1% of the general population (Maulik et al., 2011; McKenzie et al., 2016). ID is characterised by reduced cognitive function [intelligence quotient (IQ)<70] and adaptive behaviour diagnosed before 18 years of age (Tassé et al., 2016). It is estimated that 40% of ID cases can be attributed to genetic causes (Posada de La Paz et al., 2017). To date, >2000 genes have been implicated in ID aetiology (Firth and Wright, 2011; Martin et al., 2019) and 141 of these are located on the X chromosome (Tejada and Ibarluzea, 2020), which encodes a disproportionate number of genes involved in cognitive processes (Lubs et al., 2012; Skuse, 2005; Zechner et al., 2001). Indeed, due to hemizygosity for X-linked genes, more males than females have ID (Posada de La Paz et al., 2017). Based on aetiology and clinical presentation, ID comprises a heterogenous group of neurodevelopmental conditions that can either be syndromic (i.e. a part of a clinically defined set of symptoms forming a syndrome) or non-syndromic.

‘Congenital disorder of glycosylation’ (CDG) is an umbrella term for inborn defects in glycosylation enzymes that lead to ID in 60-70% of patients (Wolfe and Krasnewich, 2013). Recently, a novel type of CDG (OGT-CDG) was diagnosed in patients carrying pathogenic variants in *O*-linked β-*N*-acetylglucosamine (*O*-GlcNAc) transferase (*OGT*) (Pravata et al., 2020b). To date, 17 pathogenic OGT-CDG variants have been identified through whole-genome sequencing, and several of these have been characterised clinically and biochemically (Pravata et al., 2019, 2020a; Selvan et al., 2018; Vaidyanathan et al., 2017; Willems et al., 2017). In addition to ID and delayed achievement of developmental milestones, patients with OGT-CDG commonly present with musculoskeletal problems, brain malformations, eye abnormalities, dysmorphic features and language problems (Pravata et al., 2020b). Furthermore, heart anomalies, immune system defects, genital abnormalities and digestive problems have also been reported in individual patients. OGT-CDG thus appears to affect the development and function of multiple organ systems. The mechanisms through which OGT variants mediate ID remain to be established, although several hypotheses have been put forward (Pravata et al., 2020b).

OGT is a 117 kDa enzyme encoded by the *OGT* gene located on the X chromosome and expressed ubiquitously in human tissues (Kreppel et al., 1997; Lubas et al., 1997). OGT catalyses *O*-GlcNAcylation, an essential, dynamic modification of nucleocytoplasmic proteins with a single *O*-linked GlcNAc sugar moiety on serine and threonine residues (Kreppel et al., 1997; Torres and Hart, 1984). As opposed to other post-translational modifications that are generally mediated by protein families, *O*-GlcNAcylation is controlled by only two enzymes with opposing function, OGT and *O*-GlcNAc hydrolase (OGA), which attach and remove the GlcNAc moiety, respectively (Gao et al., 2001; Wells et al., 2002). The sugar nucleotide donor for *O*-GlcNAcylation, UDP-GlcNAc, is synthesised from glucose by the hexosamine biosynthetic pathway and, thus, *O*-GlcNAcylation is thought to serve as a nutrient sensor (Hart, 2015; Medford et al., 2013). Since 1984, when *O*-GlcNAcylation was first discovered in lymphocytes (Torres and Hart, 1984), modification of *O*-GlcNAc proteins has been shown to be ubiquitous across all human tissues (Wulff-Fuentes et al., 2021). *O*-GlcNAcylation has been implicated in a range of biological mechanisms such as cellular metabolism (Slawson et al., 2010), gene expression and regulation (Krause et al., 2018; Sakabe et al., 2010; Streubel et al., 2017; Tan et al., 2021), cell division (Drougat et al., 2012; Lefebvre et al., 2004; Leturcq and Lefebvre, 2017; Sakabe and Hart, 2010) and stem cell differentiation (Elena-Herrmann et al., 2020; Hao et al., 2019; Zhang et al., 2019). Moreover, *O*-GlcNAcylation is considered essential for survival as embryonic ablation of *Ogt* in mice (Shafi et al., 2000) and zebrafish (Webster et al., 2009) is lethal, whereas loss of OGA activity leads to perinatal death in mice (Keembiyehetty et al., 2015; Muha et al., 2021; Yang et al., 2012).

OGT is composed of 13.5 N-terminal tetratricopeptide repeats (TPRs), which mediate substrate recognition and protein-protein interactions (Iyer and Hart, 2003; Jínek et al., 2004; Rafie et al., 2017), and a globular catalytic domain, which catalyses *O*-GlcNAcylation. In addition to acting as an essential glycosylation enzyme, OGT also proteolytically activates the transcriptional co-regulator host cell factor 1 (HCF1, encoded by *HCFC1*) (Capotosti et al., 2011). HCF1 is heavily glycosylated and cleaved by OGT in the same active site; however, glycosylation and proteolysis occur through separate mechanisms (Kapuria et al., 2018; Lazarus et al., 2013). Data suggest that HCF1 might bind a large proportion of promoters in the human genome, acting as an important cell cycle progression and mitochondrial biogenesis regulator (Michaud et al., 2013). Interestingly, *HCFC1* is itself a well-known X-linked ID (XLID) gene, mutations of which are thought to alter neural stem cell maintenance and differentiation (Castro et al., 2020; Huang et al., 2012; Jolly et al., 2015).

There is growing evidence to suggest that *O*-GlcNAcylation is essential for embryonic stem cell (ESC) maintenance (Shafi et al., 2000) and embryogenesis (Jang et al., 2012; Shafi et al., 2000; Webster et al., 2009; Yang et al., 2012; Zhang et al., 2019; Zhu et al., 2020). Cellular *O*-GlcNAcylation decreases during neuronal differentiation (Liu et al., 2012), suggesting a link between dynamic *O*-GlcNAc cycling and tissue development. Furthermore, OGT *O*-GlcNAcylates transcription factors that play an important role in pluripotency and stem cell function, including octamer binding transcription factor 4 (Oct4, encoded by *Pou5f1*) (Constable et al., 2017), sex determining region Y (SRY)-box 2 (Sox2) and signal transducer and activator of transcription 3 (STAT3) (Li et al., 2017). STAT3 is a member of the JAK/STAT signalling pathway that is activated by the pluripotency stimulus leukaemia inhibitory factor (LIF) in mouse ESCs (mESCs). Here, we report three brothers affected by OGT-CDG that carry a novel OGT variant, OGT^C921Y^, with a mutation in the catalytic core of OGT, which is absent in their unaffected brother. The variant has not been previously reported, neither in the healthy population (gnomAD database) nor in patients (Human Gene Mutation Database). We show that the OGT^C921Y^ variant possesses decreased glycosyltransferase activity *in vitro* and in an mESC model. Strikingly, a knock-in of OGT^C921Y^ results in abrogated self-renewal in mESCs as shown by decreased expression of alkaline phosphatase (ALP), Oct4 and Sox2 during a clonogenic assay. These results suggest that the role of OGT in ESC self-renewal and pluripotency might contribute to clinical signs seen in OGT-CDG patients.

## RESULTS

### Three brothers with ID carry an inherited catalytic OGT variant that is absent in their unaffected brother

**Fig. 1.**
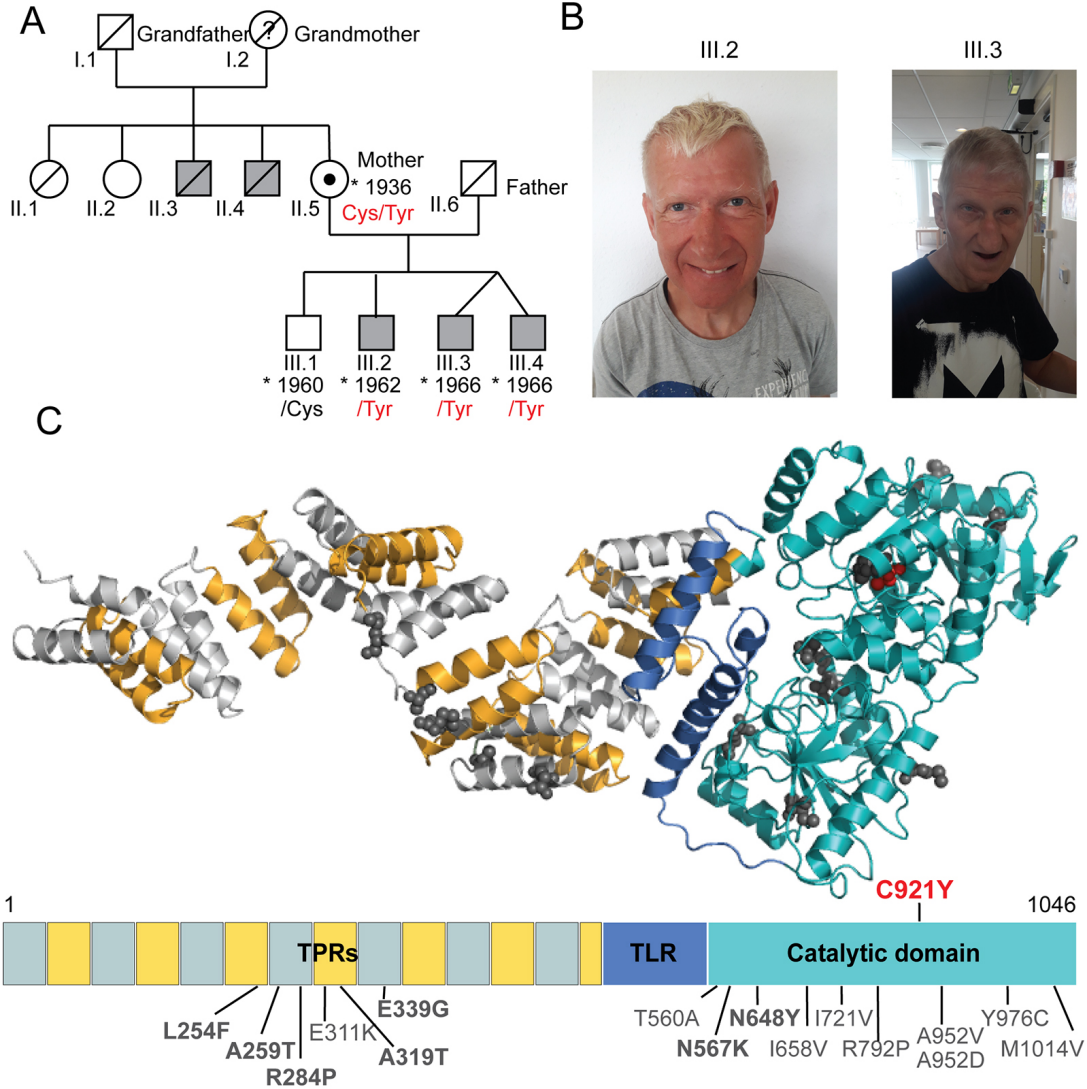
**Brothers with intellectual disability carry an inherited variant in the catalytic core of OGT that is absent in their unaffected brother.** (A) Pedigree of the affected patients carrying the OGT^C921Y^ variant. Grey squares denote individuals affected by intellectual disability. Where available, details of the OGT variant at position 921 are provided. The black dot indicates a female carrier of the OGT^C921Y^ variant. (B) Facial photographs of two brothers carrying the OGT^C921Y^ variant with the corresponding family tree annotation. Patient III.2 is depicted at the age of 58 and patient III.3 is depicted at the age of 54. (C) 3D model of full-length human OGT and the corresponding linear domain structure. Tetratricopeptide repeats (TPRs) are depicted in alternating grey and yellow, the tetratricopeptide repeat-like domain (TLR) is in dark blue and the catalytic core is in cyan. Previously known OGT variants leading to intellectual disability and their corresponding residues are highlighted in dark grey. OGT^C921Y^ is highlighted in red. The 3D model was produced using the structures of the OGT catalytic core [Protein Data Bank (PDB) code: 5C1D] and the TPR domain (PDB code: 1W3B).

Four male siblings were born to a healthy non-consanguineous couple, shown as family members II.5 and II.6 on the family pedigree (Fig. 1A). The first child was born in 1960 and is healthy. The second child, currently a 58-year-old male shown as proband III:2 on the family pedigree, was born at 38 weeks of gestation after a normal pregnancy. Birth weight was 2950 g (2nd-9th centile) and birth length was 52 cm (50th-75th centile). Apgar scores at birth are unknown. There were no concerns following birth, and the patient was discharged. Following the first 2 years of life, he showed delay in reaching developmental milestones, especially in areas of speech and language development. He learned his first words at the age of 14 months, and he is now able to speak in short sentences with five to seven words and understand simple instructions. At the age of 12 months, he could walk and reach out with palmar grasp, transfer objects and put them into his mouth. Later in life, the patient presented with autistic features. He attended a special needs school, and he currently lives in a sheltered home. He underwent an operation for an inguinal hernia at the age of 48 years. Brain MRI was never performed. He has experienced at least two generalised tonic clonic seizures. The first seizure occurred around the age of 40 years. At seizure onset, his electroencephalogram (EEG) showed background slowing, but without any clear interictal epileptiform abnormalities. After the second seizure, a daily treatment with oxcarbazepine was prescribed and he has since had a good seizure control. Dysmorphic features include an oval face, a narrow, long and pear-shaped nose with a high nasal bridge, a thin upper lip, a high arched palate, large ears, sparse eyebrows, and thin and short fingers with distal squaring (Fig. 1B; Fig. S1). At the age of 58, the patient has osteoporosis and scoliosis. He has been diagnosed with both a short stature (166 cm, −1.48 s.d.) and a head circumference of 55.3 cm (+0.12 s.d.). Cranial nerve examination was normal. Limb examination showed neither rigidity nor tremors. The power in the limbs was five out of five [Medical Research Council (MRC) Scale for Muscle Strength] and the deep tendon reflexes were normal. He presented with a slow, shuffling gait.

The mother later gave birth to monozygotic male twins, here referred to as twin 1 and twin 2 (III:3 and III:4 in pedigree), at 38 weeks of gestation following a normal pregnancy. Conception was unassisted. Twin 1 (III:3) weighed 2150 g at birth (<0.4th centile) and had a birth length of 48 cm (2nd-9th centile), whereas twin 2 (III:4) weighed 1650 g (<0.4th centile) and measured 46 cm (0.4^th^-2nd centile). Apgar scores are unavailable. Although twin 1 was breastfed and discharged within a week following the birth, twin 2 was tube fed for 2 weeks. Similar to proband III:2, the twins also showed delay in reaching developmental milestones, especially in areas of speech and language development. Twin 2 never learned to speak and twin 1 is able to pronounce a few words. The twins have autism and communicate using simple signs and by pointing. They are able to understand very simple instructions. Both make high-pitched sounds, have repetitive mannerisms and clap their hands together if excited. In addition, twin 2 has self-injurious behaviour. Their motor development was also affected; although both twins learned to walk by the age of 18 months, they were diagnosed with significant gross motor difficulties and clumsiness. They have attended a special needs nursery and school and they currently live in a sheltered home. Neither of the twins underwent a brain MRI. Although twin 1 has not had an epileptic seizure, twin 2 experienced his first generalised tonic clonic seizure at around 40 years of age. Over the subsequent 11 years, he had a total of four identical epileptic seizures.

At the time of the first seizure, the EEG in twin 2 showed background slowing, but without any clear interictal epileptiform abnormalities. After the second seizure, a daily treatment with carbamazepine was prescribed. Data on dysmorphic features from childhood were not available, but both twins currently present with an oval face, up-slanting palpebral fissures, a narrow, long and pear-shaped nose with a high nasal bridge, a thin upper lip, hypotrichosis and thin fingers (Fig. 1B; Fig. S1). Twin 2 has also been diagnosed with scoliosis, short stature (165 cm, −2.1 s.d.) and osteoporosis. In addition, twin 2 does not have any rigidity or tremors of the extremities but has a slow shuffling gait. Limited clinical data is available for twin 1.

The three brothers III:2, III:3 and III:4 thus appeared to have an inherited developmental defect that was not present in their eldest brother (III:1). Family history revealed that two maternal uncles (II.3 and II.4) also suffered from ID and were wheelchair bound at an older age, suggesting an X-linked recessive inheritance pattern. Whole-genome sequencing of patient III:2 and subsequent Sanger analysis of the *OGT* gene in samples from all three remaining brothers and their mother were performed. These analyses revealed that the affected siblings III:2, III:3 and III:4 carried a guanine-to-adenine substitution at nucleotide 2762 (c.2762G>A) in the *OGT* gene, which translates as a cysteine-to-tyrosine substitution at position 921 in OGT [c.2762G>A p.(C921Y), NM_181672.2, OGT^921Tyr^] (Fig. 1C) inherited from their heterozygous mother (OGT^921Cys/Tyr^). This OGT variant has not been previously reported and it is classified as likely pathogenic according to the American College of Medical Genetics and Genomics (ACMG) criteria (criteria PM1, PM2, PP1, PP2, PP3 and PP4) (Richards et al., 2015). The OGT^C921Y^ variant was absent in the unaffected brother (II:1, OGT^921Cys^), suggesting that this single point mutation in *OGT* causes ID in patients II:2, II:3 and II:4. Thus, we have identified three brothers with ID who carry an inherited catalytic OGT variant that is absent in the unaffected brother. The clinical observations are consistent with previous clinical descriptions of OGT-CDG patients and add considerable knowledge to the currently limited information on adult OGT-CDG patients (Pravata et al., 2020b). For example, despite an established association between decreased *O*-GlcNAcylation and Alzheimer's disease (Liu et al., 2004, 2009; Park et al., 2021), these patients do not present with signs of age-related neurodegenerative disorders.

### OGT^C921Y^ is defective in glycosyltransferase activity towards protein substrates *in vitro*

**Fig. 2.**
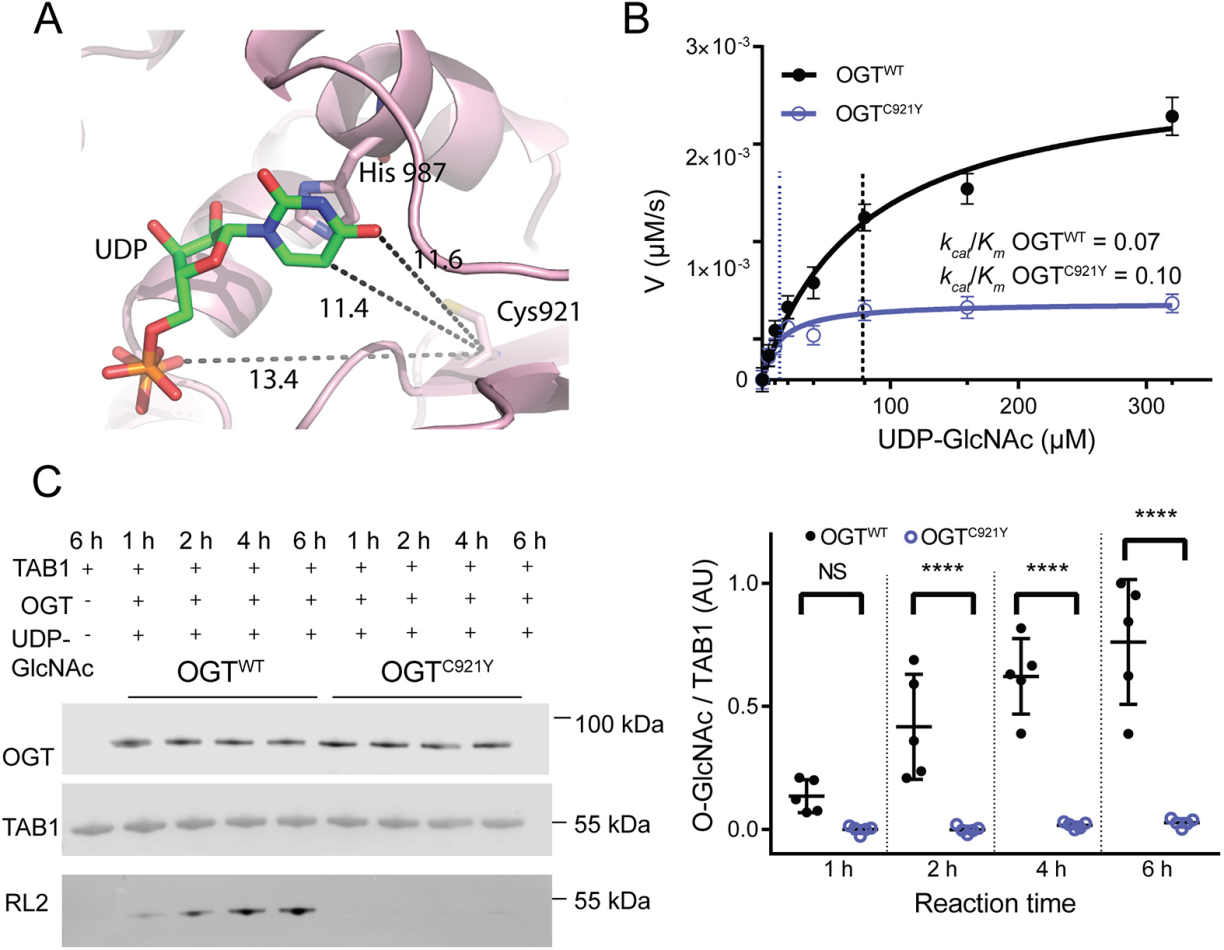
**OGT^C921Y^ is defective in glycosyltransferase activity towards protein substrates *in vitro.*** (A) Magnified view of the catalytic domain of OGT. UDP is shown as sticks. Distances (in Å) between the C921 residue and UDP are highlighted as dotted lines (11.4 Å from the C921 carbon α to the uracil ring in green, 13.4 Å from the C921 carbon α to the pyrophosphate linker in orange). (B) Michaelis–Menten kinetics of recombinant full-length OGT^WT^ and OGT^C921Y^ incubated with the acceptor peptide (Ac-KENSPAVTPVSTA-NH2) and varying concentrations of UDP-GlcNAc. V, reaction velocity. *n*=3 repeats, each consisting of three technical replicates. (C) Immunoblot detection of TAB1 *O*-GlcNAcylation by recombinant OGT^WT^ and OGT^C921Y^ (323-1041 aa) over the course of 1, 2, 4 and 6 h *in vitro*. The corresponding immunoblot signal quantification is shown on the right. *n*=5 reactions using the same batch of recombinant protein. Repeated-measures two-way ANOVA with Sidak's multiple comparison test was used to determine significance: 0 h, *P=*0.14; 2 h, *P*<0.0001; 4 h, *P*<0.0001; 2 h, *P*<0.0001. Error bars represent s.d. AU, arbitrary units. NS, not significant; ****P<0.0001.

Disrupted OGT stability and folding caused by pathogenic missense variants could contribute to OGT-CDG pathophysiology (Pravata et al., 2020b). Therefore, the effect of the C921Y substitution on the OGT protein structure was investigated. Although we were able to produce this variant in recombinant form from an *Escherichia coli* expression system, we were unable to grow crystals for structural analysis, unlike several of the previously reported variants (Gundogdu et al., 2018; Pravata et al., 2019, 2020a; Selvan et al., 2017; Vaidyanathan et al., 2017). Nevertheless, analysis of the wild-type (WT) OGT structure reveals that C921 resides in the catalytic domain of OGT, proximal to the UDP-GlcNAc binding site (∼13 Å; Fig. 2A). The mutation of C921 to a bulky tyrosine residue would disrupt the C845-C921 disulphide bridge found in OGT^WT^. Furthermore, C921Y could perturb the position of H911, which in the WT enzyme establishes π−π stacking interactions with the UDP uracil ring (Fig. S2A,B). The mutation could also affect N935, induce the loss of the Y851-UDP interaction and promote the generation of a new interaction between Y931 and UDP (Fig. S2A,B). Thus, the C921Y substitution could affect protein stability, interfere with catalysis and/or alter binding of UDP-GlcNAc to OGT.

We next investigated whether the C921Y mutation disrupts the stability of recombinant OGT [323-1044 amino acids (aa)] *in vitro* using a thermal denaturation assay. No change in melting temperature (*T*_m_) between truncated OGT^WT^ (*T*_m_=45±1°C; mean±s.d.) and OGT^C921Y^ (*T*_m_=45±1°C) was observed (*n*=3 replicates, each consisting of three technical repeats) (Fig. S3). Next, we examined the impact of the C921Y mutation on the glycosyltransferase activity of full-length OGT. We first used a steady-state kinetics assay with varying concentrations of the sugar donor UDP-GlcNAc against an established acceptor peptide (Ac-KENSPAVTPVSTA-NH_2_; Pathak et al., 2015) (Fig. 2B). The maximal reaction velocity (*V*_max_) and the Michaelis–Menten constant (*K*_m_) were reduced for OGT^C921Y^ [*V*_max_=(0.70±0.01)×10^−3^ µmol/s, *K*_m_=14±4 µM] compared to those for OGT^WT^ [*V*_max_=(2.8±0.2)×10^−3^ µmol/s, *K*_m_=78±15 µM]. However, the catalytic efficiencies (*k*_cat_/*K*_m_) of the mutant and WT enzyme were similar (OGT^WT^, *k*_cat_/*K*_m_=0.07; OGT^C921Y^, *k*_cat_/*K*_m_=0.10; *n*=3 replicates, each consisting of three technical repeats). Thus, the catalytic activity of OGT^C921Y^ towards a peptide substrate is intact compared to that of OGT^WT^.

We next evaluated the effects of the mutation on OGT activity towards TAK1-binding protein 1 (TAB1), a well-characterised protein substrate (Pathak et al., 2012; Rafie et al., 2017), revealing a loss of OGT^C921Y^ catalytic activity compared to that of OGT^WT^ (Fig. 2C). Western blot analysis of TAB1 *O*-GlcNAcylation (indicated by the RL2 antibody) showed that the time-dependent increase in TAB1 *O*-GlcNAc signal produced by OGT^WT^ was absent in the OGT^C921Y^-catalysed reaction (Fig. 2C). The mean level of TAB1 *O*-GlcNAcylation catalysed by OGT^C921Y^ after 6 h was 28-fold lower than that catalysed by OGT^WT^ (two-way ANOVA with Sidak's multiple comparison testing, *P*<0.0001).

These data reveal that although OGT^C921Y^ catalytic activity towards protein substrates is abrogated, it appears intact towards peptides, implying that the disruption of substrate interactions with the catalytic domain of OGT caused by the C921Y mutation prevents accommodation of large acceptor substrates. Interestingly, this suggests a previously unappreciated role of catalytic domain residues in recognition of protein substrate features beyond the site of *O*-GlcNAc modification. Taken together, these data show that OGT^C921Y^ is defective in glycosyltransferase activity towards protein substrates *in vitro*.

### OGT^C921Y^ disrupts *O*-GlcNAc homeostasis in undifferentiated mESCs

To investigate the impact of the OGT^C921Y^ variant on *O*-GlcNAc cycling, we engineered male OGT^C921Y^ mESC lines using CRISPR/Cas9. The murine and human OGT protein sequences are identical in the mutated region (Fig. S4A). Three independent CRISPR/Cas9 lines carrying OGT^C921Y^ were generated through clonal expansion from three distinct founder cells (Fig. S4B). OGT^C921Y^ mESC lines were morphologically identical to wild-type (or OGT^WT^) cells and their cell cycle was not affected (Fig. S5). mRNA and protein levels of the key pluripotency factors Oct4 and Sox2 remained the same as in OGT^WT^ mESC (Fig. S6). All the following experiments were performed using three different cell clones per genotype and repeated over multiple passages, unless stated otherwise.

**Fig. 3.**
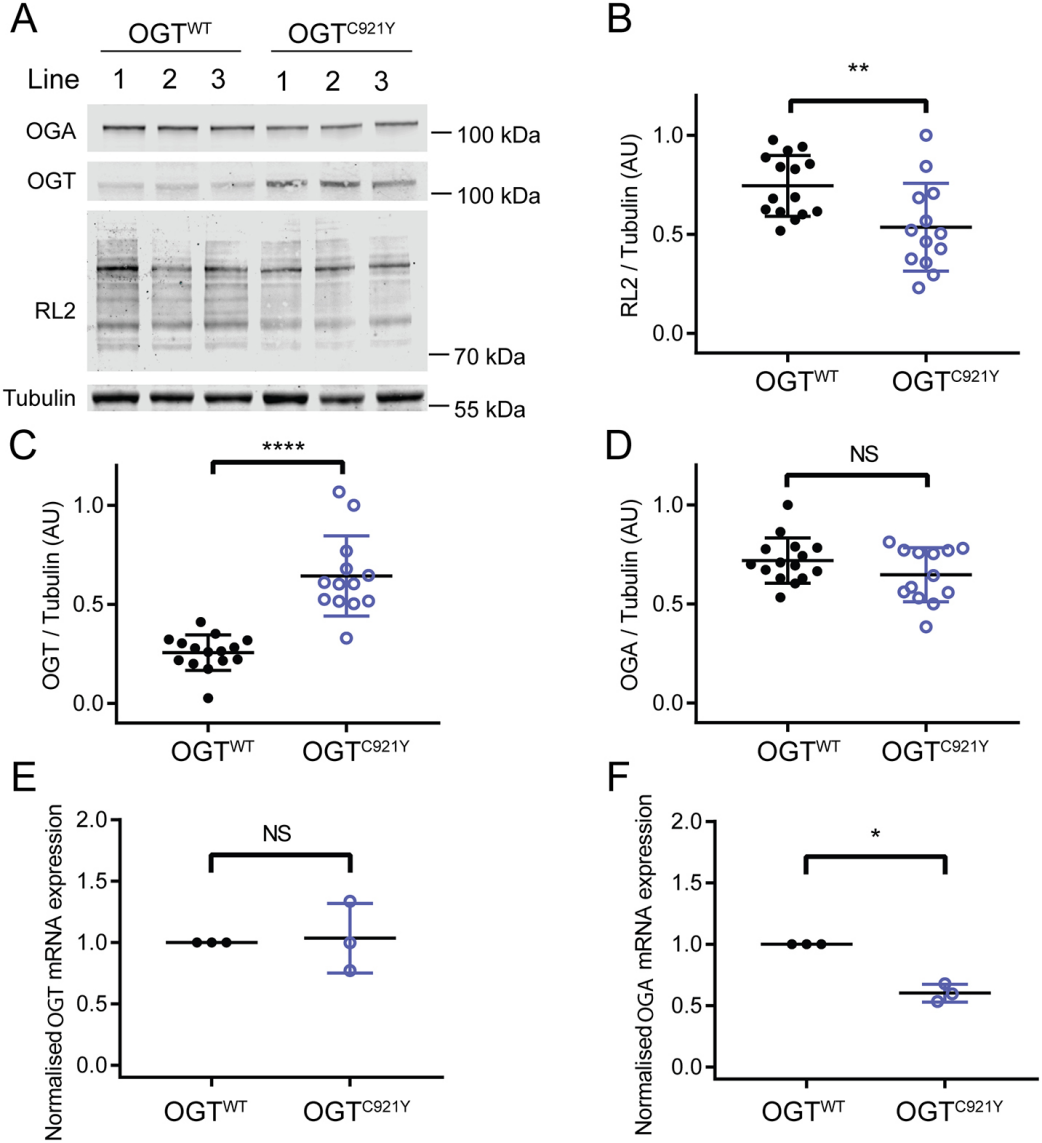
**The OGT^C921Y^ variants disrupt the *O*-GlcNAcylation machinery in undifferentiated mESCs.** (A) Immunoblots showing detection of *O*-GlcNAcylation (RL2 antibody), OGT and OGA in mESCs carrying OGT^WT^ or the OGT^C921Y^ variant. (B-D) Quantification of RL2 (B), OGT (C) and OGA (D) immunoblot signals. RL2, OGT and OGA signals were normalised to those of tubulin. Quantification in B-D is based on results obtained from three different cell clones per genotype and repeated over four to five passages per clone. OGT^WT^, *n*=15; OGT^C921Y^, *n*=13; unpaired two-tailed *t*-test. *P*=0.007 (B); *P*<0.0001 (C); *P*=0.14 (D). (E,F) RT-PCR analysis of *Ogt* (E) and *Oga* (F) mRNA levels normalised to those of *Gapdh*, 18S (*Rn18s*) and *Actb*. Data points representing the mean expression calculated from three separate RT-PCR runs are shown. Each RT- PCR run was set up using several OGT^WT^ and OGT^C921Y^ as biological replicates. Unpaired two-tailed *t*-test. *P*=0.84 (E); *P*=0.0007 (F). Error bars represent s.d. AU, arbitrary units. NS, not significant; *P<0.05; **P<0.01; ****P<0.0001.

Western blot analyses from pluripotent OGT^C921Y^ mESCs revealed a significant decrease in global *O*-GlcNAc levels compared to OGT^WT^ cells (Fig. 3A,B; OGT^WT^, *n*=15 biological replicates; OGT^C921Y^, *n*=13 biological replicates; unpaired two-tailed *t*-test, *P*=0.0072), suggesting an alteration in *O*-GlcNAc homeostasis. We detected a significant upregulation of OGT protein levels in OGT^C921Y^ cells compared to OGT^WT^ cells (Fig. 3A,C; OGT^WT^, *n*=15 biological replicates; OGT^C921Y^, *n*=13 biological replicates; unpaired two-tailed *t*-test, *P*<0.0001). However, given the decreased global *O*-GlcNAcylation levels (indicated by the RL2 antibody) in the mutant mESC lines, these data suggest that despite being present at higher levels, OGT^C921Y^ glycosyltransferase activity is not sufficient to maintain *O*-GlcNAc homeostasis in mESCs. OGA protein levels were unchanged in the mutant cell line (Fig. 3A,D; OGT^WT^, *n*=15 biological replicates; OGT^C921Y^, *n*=13 biological replicates; unpaired two-tailed *t*-test, *P*=0.14). In contrast, previously characterised OGT-CDG variants showed a reduction in OGA protein levels when modelled in mESCs or patient-derived lymphoblastoids, and OGT variant levels remained unchanged compared to those of OGT^WT^ (Pravata et al., 2019; Pravata et al., 2020a; Vaidyanathan et al., 2017).

We next evaluated *Ogt* and *Oga* expression by real-time PCR (RT-PCR) analysis of three biological replicates with up to three OGT^C921Y^ and OGT^WT^ lines. These experiments showed that *Ogt* mRNA expression is unchanged in OGT^C921Y^ lines compared to that in OGT^WT^ lines (Fig. 3E; *n*=3 RT-PCR runs, each consisting of two to three biological replicates; two-way ANOVA, *P*=0.82), implying that OGT might be stabilised at the protein level in OGT^C921Y^ mESCs, possibly through decreased protein degradation in cultured cells. Qian et al. (2018) revealed a bidirectional feedback mechanism between *Ogt* and *Oga* at the transcriptional level in primary mouse hepatocytes using an overexpression system. Furthermore, OGT has been shown to direct the transcriptional repressor Sin3A-HDAC1 complex to the *OGA* promoter in OGT-CDG patient-derived lymphoblastoids, thus modulating *OGA* expression (Vaidyanathan et al., 2017). Indeed, *Oga* mRNA levels were significantly decreased in OGT^C921Y^ mESCs (Fig. 3F; *n*=3 RT-PCR runs, each consisting of two to three biological replicates; unpaired two-tailed *t*-test, *P*=0.0007) even though protein levels were unchanged. Taken together, these data reveal that OGT^C921Y^ disrupts *O*-GlcNAc homeostasis in pluripotent mESCs despite compensatory changes in the levels of OGT protein and *Oga* gene expression.

### HCF1 processing is unchanged in pluripotent OGT^C921Y^ mESCs

**Fig. 4.**
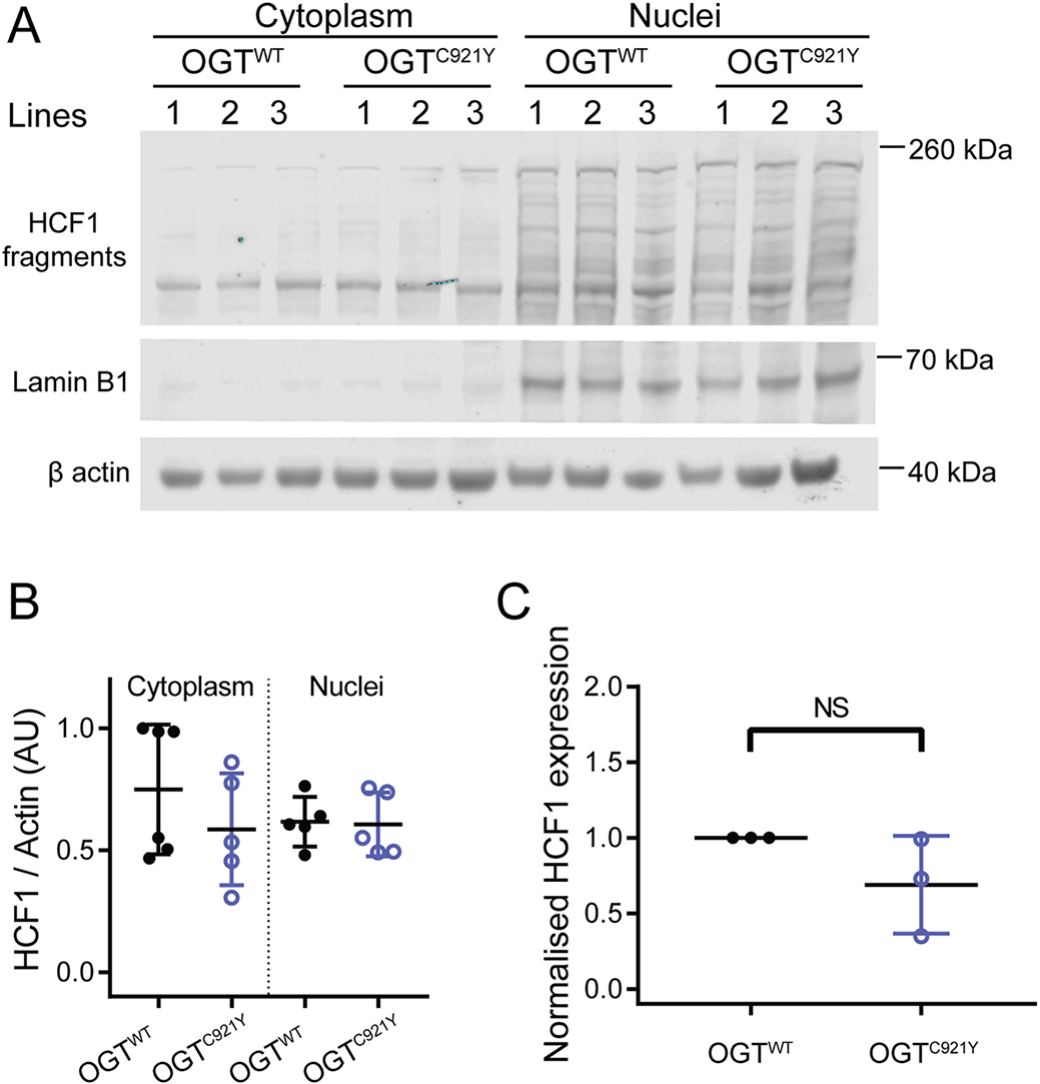
**HCF1 processing by undifferentiated OGT^WT^ and OGT^C921Y^ in mESCs.** (A) Immunoblot of HCF1 proteolytic fragments in the cytoplasm and nucleus of mESCs harbouring either OGT^WT^ or the OGT^C921Y^ variant. Lamin B1 was used as a marker for the nuclear fraction. This experiment was performed using three cell clones per genotype and repeated over two passages. (B) Quantification of HCF1 signal. HCF1 signals were normalised to those of β-actin. *n*=6 biological replicates. One-way ANOVA with Tukey’s comparison test; cytoplasmic fraction, *P*=0.99; nuclear fraction, *P*=0.54. (C) RT-PCR analysis of *Hcf1* mRNA expression levels normalised to those of *Gapdh*, 18S (*Rn18s*) and *Actb*. Data points representing the mean expression calculated from three separate RT-PCR runs are shown. Each RT-PCR run was set up using several OGT^WT^ and OGT^C921Y^ lines as biological replicates. Unpaired two-tailed *t*-test, *P*=0.172. Error bars represent s.d. AU, arbitrary units. NS, not significant.

In addition to catalysing *O*-GlcNAcylation, OGT also proteolytically processes and activates the transcriptional coregulator HCF1 (Capotosti et al., 2011). HCF1 is encoded by *HCFC1*, which itself has been reported to be an ID-related gene (Huang et al., 2012; Wongkittichote et al., 2021). Biochemical analysis of two previously reported OGT-CDG variants showed decreased HCF1 proteolysis (Pravata et al., 2019; Willems et al., 2017). HCF1 proteolysis occurs within the same active site of OGT as *O*-GlcNAcylation (Kapuria et al., 2016; Lazarus et al., 2013). Given the proximity of the C921Y substitution to the OGT active site (Fig. 2A; Fig. S1) and the impact of the C921Y variant on OGT glycosyltransferase activity, we investigated whether this variant leads to deficient HCF1 *O*-GlcNAcylation and processing. First, we performed an *in vitro* HCF1 repeat 1 (HCF1rep1) cleavage assay using full-length recombinant OGT^WT^ or OGT^C921Y^ (Fig. S7). The uncleavable mutant HCF1rep1^E10D^ was used in this assay as a negative control to allow us to distinguish between non-specific HCF1 degradation and bona fide proteolytic products (Fig. S7). In agreement with the loss of *O*-GlcNAcylation activity on TAB1, we observed decreased levels of *O*-GlcNAcylation of HCF1rep1^WT^ and HCF1rep1^E10D^ by OGT^C921Y^ (Fig. S7). However, both the WT and mutant OGT were able to catalyse the formation of HCF1 proteolytic products (Fig. S7). To corroborate this further, we investigated HCF1 cleavage in the mESC model (Fig. 4A). As HCF1 translocates to the nucleus, the abundance of HCF1 proteolytic fragments was inspected in both the cytoplasmic and nuclear fractions of undifferentiated mESCs (Fig. 4A). We observed no difference in HCF1 signal between OGT^WT^ and OGT^C921Y^ cells in either of the cellular compartments (Fig. 4B; *n*=6 biological replicates; one-way ANOVA with Tukey’s comparison test; cytoplasmic fraction, *P*=0.99; nuclear fraction, *P*=0.54). RT-PCR analysis revealed that *Hcf1* mRNA levels remained stable in OGT^C921Y^ mESCs (Fig. 4C; *n*=3 RT-PCR runs, each consisting of two to three biological replicates; unpaired two-tailed *t*-test, *P*=0.172). Taken together, these data show that HCF1 processing is unchanged in undifferentiated OGT^C921Y^ mESCs.

### OGT^C921Y^ abrogates mESC self-renewal capacity

Analogous to previously reported OGT-CDG mutations (Pravata et al., 2020b), patients carrying the OGT^C921Y^ variant present with a broad array of phenotypes, including developmental delay, brain abnormalities and musculoskeletal defects. Embryonic development and patterning are crucially dependent on the ability of the inner cell mass of the early embryo to respond to the presence of differentiation stimuli or the absence of self-renewal stimuli. The ability of the inner cell mass as well as cultured ESCs to develop into the three primary germ cell layers is called pluripotency. Pluripotent cells maintain their identity through self-renewal. In the context of embryonic development, self-renewal and differentiation are meticulously orchestrated by various signalling molecules. In cell culture, mESCs are commonly propagated through LIF supplementation in the growth medium (Smith et al., 1988; Williams et al., 1988). Upon LIF withdrawal, mESCs lose expression of pluripotency markers such as Oct4 and Nanog, colonies acquire a flattened morphology, and cells begin to differentiate (Chen et al., 2015; Cherepkova et al., 2016; He et al., 2017). *O*-GlcNAcylation has been previously shown to be important for core and auxiliary pluripotency factor function (Constable et al., 2017; Hao et al., 2019; Jang et al., 2012; Kim et al., 2021; Myers et al., 2016). Therefore, we hypothesised that the OGT^C921Y^ variant might impact the ability of stem cells to maintain an undifferentiated state. To test this hypothesis, we assayed colony formation and maintenance of stemness upon LIF withdrawal.

**Fig. 5.**
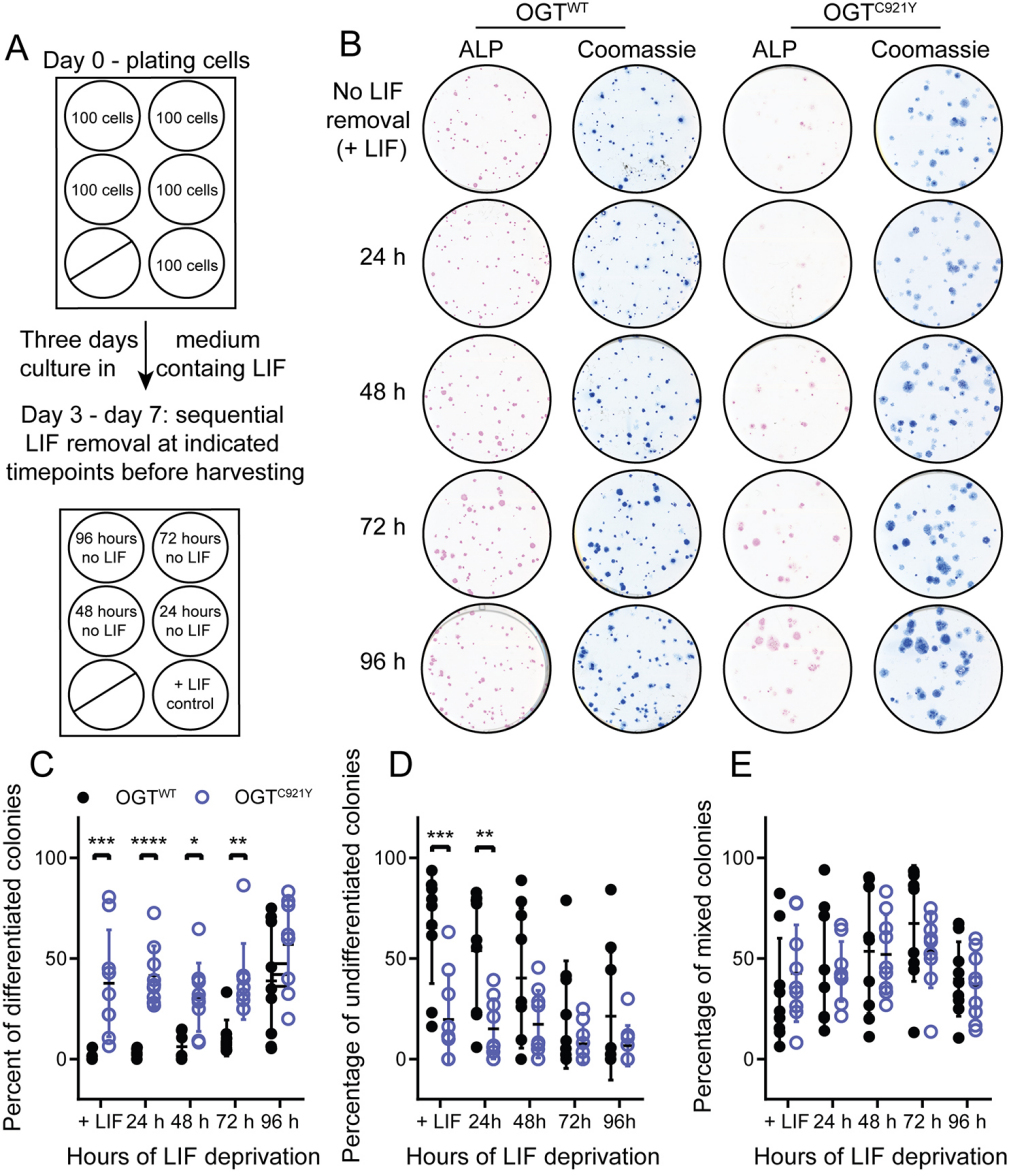
**The OGT^C921Y^ variant leads to increased differentiation in response to LIF withdrawal in mESCs.** (A) Schematic representation of the experimental set up. This experiment was performed using three cell clones per genotype and repeated over three passages. (B) Scans of alkaline phosphatase (ALP, pink)-stained colonies of OGT^WT^ or OGT^C921Y^ mESCs at different time points after LIF withdrawal. Each well was re-stained by Coomassie Blue (blue) to reveal ALP-negative colonies. (C) Plot of percentages of differentiated colonies in OGT^WT^ and OGT^C921Y^ mESCs. *n*=9 biological replicates; two-way ANOVA; +LIF, *P*=0.0001; 24 h, *P*=0.005; 48 h, *P*=0.21; 72 h, *P*=0.68; 96 h, *P*=0.67. (D) Plot of percentages of undifferentiated colonies in OGT^WT^ and OGT^C921Y^ mESCs. *n*=9 biological replicates; two-way ANOVA; +LIF, *P*=0.0006; 24 h, *P*<0.0001; 48 h, *P*=0.017; 72 h, *P*=0.004; 96 h, *P*=0.13. (E) Plot of percentages of mixed colonies in OGT^WT^ and OGT^C921Y^ mESC. *n*=9 biological replicates, two-way ANOVA; all *P*-values are non-significant. Error bars represent s.d. *P<0.05; **P<0.01; ***P<0.001; ****P<0.0001.

OGT^C921Y^ and OGT^WT^ mESCs were subjected to clonogenic conditions in the presence of LIF (positive control) or followed by LIF withdrawal for 24-96 h before fixation and ALP staining (Fig. 5A). High expression of ALP is considered to be a marker of pluripotency, with ALP staining visualising pluripotent colonies (Štefková et al., 2015). The ALP-stained colonies were examined under a light microscope and classified into three categories (undifferentiated, mixed and differentiated), based on the intensity of the ALP stain and colony morphology. Examples of colony scoring are shown in Fig. S8. In the OGT^WT^ cell lines, the percentage of differentiated colonies increased after LIF withdrawal (Fig. 5B,C; *n*=9 biological replicates; one-way ANOVA with Dunnett's multiple comparison test, *P*<0.0001) and the percentage of undifferentiated colonies significantly decreased with time of culture in the absence of LIF (Fig. 5B,D; *n*=9 biological replicates; one-way ANOVA with Dunnett's multiple comparison test, *P*=0.012). However, the percentages of undifferentiated and differentiated mutant OGT^C921Y^ colonies did not change significantly in relation to the length of LIF deprivation (Fig. 5B-D; *n*=9 biological replicates, one-way ANOVA with Dunnett's multiple comparison test, *P*=0.10 and *P*=0.29, respectively). We noted that OGT^C921Y^ mESCs produced a significantly higher percentage of differentiated colonies than OGT^WT^ mESCs at every LIF withdrawal time point apart from 96 h (Fig. 5B,C; *n*=9 biological replicates; two-way ANOVA; 24 h, *P*<0.0001; 48 h, *P*=0.02; 72 h, *P*=0.004; 96 h, *P*=0.13). Remarkably, there was a significantly increased number of OGT^C921Y^ differentiated colonies even in the presence of LIF (*n*=9 biological replicates; two-way ANOVA, *P*=0.0001) compared to differentiated OGT^WT^ colonies. The number of mixed colonies remained constant over the course of the assay in both OGT^WT^ and OGT^C921Y^ mESCs. Furthermore, the overall number of colonies formed by OGT^C921Y^ mESCs was significantly lower than for OGT^WT^ mESCs (Fig. S9; *n*=45 wells scored; unpaired two-tailed *t*-test, *P*<0.0001). This observation can be explained by a decrease in the clonogenic potential of OGT^C921Y^ cells compared to OGT^WT^ cells, and/or increased cell death following cell plating at limiting density. Lastly, OGT^C921Y^ mESCs formed colonies with a significantly larger surface area than that of OGT^WT^ cells (Fig. S9; OGT^WT^, *n*=2270 colonies measured; OGT^C921Y^, *n*=1533 colonies measured; unpaired two-tailed *t*-test, *P*<0.0001; observations were pooled together from all three independent experiments performed using three clones per genotype). Given the loss of ALP staining in OGT^C921Y^, the observed increase in surface area might reflect flattening and concomitant expansion of OGT^C921Y^ colonies due to differentiation.

**Fig. 6.**
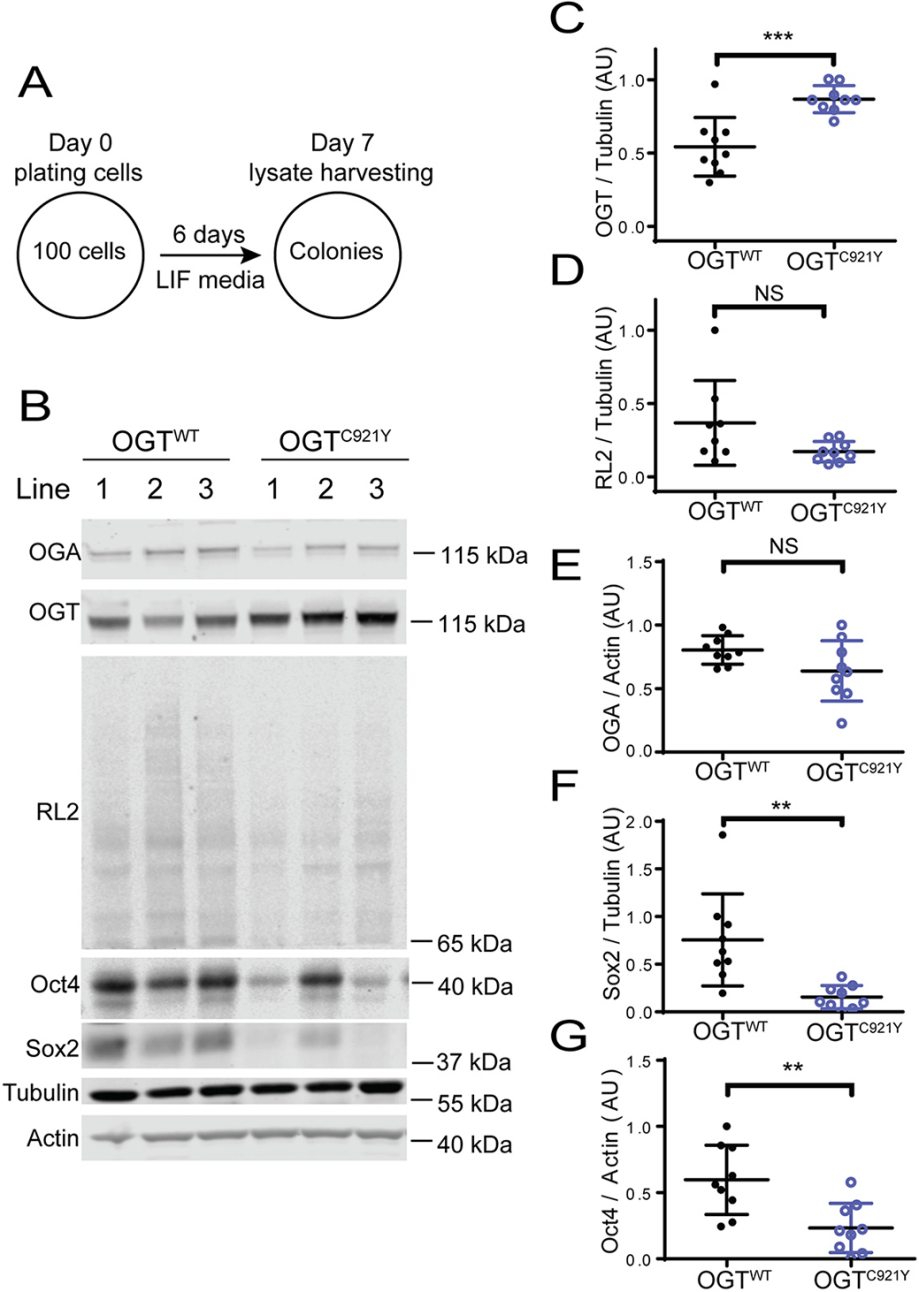
**mESCs harbouring the OGT^C921Y^ variant downregulate Sox2 and Oct4 in response to clonogenic conditions.** (A) Schematic representation of the experimental set up. The experiments were performed using three cell clones per genotype and repeated over three passages. (B) Immunoblot showing detection of *O*-GlcNAcylation (RL2 antibody), OGT, OGA, Oct4 and Sox2 in mESC lines carrying OGT^WT^ or the OGT^C921Y^ variant. Lysates were derived from cells grown under clonogenic conditions in the presence of LIF as shown in A. (C-G) Quantification of OGT (C), RL2 (D), OGA (E), Sox2 (F) and Oct4 (G) immunoblot signals. OGT, RL2 and Sox2 signals were normalised to those of tubulin; OGA and Oct4 signals were normalised to those of actin. OGT^WT^, *n*=9 biological replicates (except for RL2, where *n*=8 biological replicates); OGT^C921Y^, *n*=9 biological replicates. Unpaired two-tailed *t*-test; *P*=0.0004 (C); *P*=0.067 (D); *P*=0.077 (E); *P*=0.0023 (F); *P*=0.0038 (G). Error bars represent s.d. AU, arbitrary units. NS, not significant; **P<0.01; ***P<0.001.

To further corroborate the *O*-GlcNAcylation status and enzymatic activity of OGT^C921Y^ mESCs under clonogenic conditions, we investigated global protein *O*-GlcNAcylation levels and protein levels of key pluripotency transcription factors (Sox2 and Oct4). OGT^C921Y^ and OGT^WT^ mESCs were cultured for 7 days under clonogenic conditions in the presence of LIF (Fig. 6A). As opposed to the decreased glycosyltransferase activity of the OGT^C921Y^ variant *in vitro* and in confluent mESCs, the decrease in global *O*-GlcNAcylation in OGT^C921Y^ colonies compared to that in OGT^WT^ colonies was not significant (Fig. 6B,D; OGT^WT^, *n*=8 biological replicates; OGT^C921Y^, *n*=9 biological replicates; unpaired two-tailed *t*-test, *P*<0.07), nor was OGA (Fig. 6B,E), despite increased levels of the OGT protein (Fig. 6B,C; *n*=9 biological replicates; unpaired two-tailed *t*-test, *P*=0.0004). Previous reports indicated that global *O*-GlcNAcylation is highest in pluripotent stem cells and decreases as pluripotency is lost (Liu et al., 2012; Sheikh et al., 2020). Our observation might therefore reflect the shift from predominantly pluripotent stem cell population present in confluent mESCs propagated in LIF to a mixed population of cells present at day 6 of the clonogenic assay (Fig. 5C-E). Furthermore, western blot analysis of lysates derived from OGT^C921Y^ colonies revealed a significant decrease of Oct4 (Fig. 6B,G; *n*=9 biological replicates; unpaired two-tailed *t*-test, *P*=0.004) and Sox2 (Fig. 6B,F; *n*=9 biological replicates; unpaired two-tailed *t*-test, *P*=0.002) levels compared to those in lysates from OGT^WT^ colonies. This finding was corroborated by immunofluorescence staining of OGT^WT^ and OGT^C921Y^ colonies (Fig. S10). It is worth noting that we observed a variability in Sox2 and Oct4 protein levels among the three lines of OGT^C921Y^ mESCs, possibly stemming from the fact that each cell line is derived from a separate CRISPR/Cas9 event, therefore representing three cell populations that arose from three different founder cells. To discern whether differentiating OGT^C921Y^ mESCs assume a specific germ layer preferentially, colonies were immunolabelled for markers specific for the mesoderm (Brachyury, encoded by *Tbxt*), endoderm (Sox17) and ectoderm (Pax6). There was no difference in germ layer marker protein expression in the mutant and WT colonies (Figs S11-S14). However, this result is limited by the short timeframe of the assay. Taken together, these data show that independent of LIF signalling, OGT^C921Y^ mESCs showed reduced ALP staining under clonogenic conditions compared to that seen for OGT^WT^ colonies, suggesting defects in stem cell renewal. Furthermore, OGT^C921Y^ colonies grown in the presence of LIF showed reduced levels of Sox2 and Oct4 transcription factors, implying that OGT^C921Y^ affects self-renewal of mESC under these conditions.

## DISCUSSION

Pathogenic missense variants in the *OGT* gene co-segregate with ID in at least 17 known affected families, seven of which have been described in detail (Bouazzi et al., 2015; Pravata et al., 2019, 2020a; Selvan et al., 2018; Vaidyanathan et al., 2014; Willems et al., 2017). Through biochemical, cellular and animal model assays, recent studies revealed that these single *OGT* point mutations (hemizygous or heterozygous) underpin pathogenesis, via unknown biological mechanisms. This condition was recently classified as a distinct type of CDG, termed OGT-CDG (Pravata et al., 2020b). Here, we describe a family affected by a C921Y variant in the catalytic domain of OGT. Three male siblings with ID harbour the OGT^C921Y^ variant, whereas their brother who possesses a WT copy of *OGT* is not affected. All three affected brothers present with dysmorphic features such as a long and pear-shaped nose, as well as behavioural and language problems. These features are in line with the recently reported general clinical phenotype description of OGT-CDG patients (Pravata et al., 2020b). The severity of ID in the OGT^C921Y^ patients varied. Interestingly, the levels of ID manifestation was different even between the two twins affected by this novel *OGT* variant. Similar differences in ID severity were reported by Pravata et al. (2019) in female monozygotic twins affected by OGT-CDG.

The C921Y mutation is predicted to cause structural defects within the OGT catalytic core, albeit without measurable effects on stability *in vitro*. Interestingly, only two of the OGT-CDG variants biochemically characterised to date (OGT^N567K^ and OGT^N648Y^) did not lead to changes in OGT stability (Pravata et al., 2019, 2020a). OGT^N567K^, OGT^N648Y^ and OGT^C921Y^ are the only three described variants directly affecting the catalytic domain, whereas other OGT-CDG mutations reside in the TPR domain and result in destabilisation of the protein. Collectively, these data suggest that either loss of the functional OGT protein or disruption of OGT catalytic activity lead to the same clinical phenotype. Biochemical characterisation of additional variants affecting catalysis, solution of their crystal structures and examination of OGT localisation in OGT-CDG cells will contribute to testing this hypothesis.

Previous research has revealed a role of the OGT TPR domain in protein-protein interactions and substrate recognition (Iyer and Hart, 2003; Jínek et al., 2004; Lazarus et al., 2013; Levine et al., 2018; Pathak et al., 2015; Rafie et al., 2017), demonstrating the role of an asparagine ladder in substrate binding. Our data imply a potential role of catalytic domain residues in substrate recognition, substantiated by predicted structural changes in the proximity of the active site and a loss of glycosyltransferase activity towards a protein substrate, without loss of activity towards a peptide substrate. We also revealed that OGT activity in cultured mESCs was affected by the C921Y variant, leading to global hypo-*O*-GlcNAcylation. Decreased *O*-GlcNAcylation in cultured cells as a result of an OGT-CDG mutation has previously only been observed for the OGT^N648Y^ variant (Pravata et al., 2020a). *O*-GlcNAc homeostasis is tightly regulated by feedback loops among OGT, OGA and *O*-GlcNAc levels (Decourcelle et al., 2020; Lin et al., 2021; Muthusamy et al., 2015; Tan et al., 2021; Zhang et al., 2014). This has been shown to occur through both translational and post-translational mechanisms, for example, through the regulation of intron detention, which alters the abundance of productive transcripts of *OGT* and *OGA* (Tan et al., 2021). Unlike in OGT^C921Y^ cells, this generally manifests as downregulation of OGA at the protein level, particularly upon OGT inhibition or in OGT-CDG catalytic domain mutations (Ortiz-Meoz et al., 2015; Pravata et al., 2019, 2020a). However, reducing *O*-GlcNAcylation through inhibiting the hexosamine biosynthetic pathway has been shown to increase OGT protein levels (Lin et al., 2021). Importantly, OGA is also implicated in neurodevelopment (Olivier-Van Stichelen et al., 2017) and cognitive functioning (Muha et al., 2020), both in model animals and potentially in humans (as determined by a genome-wide association study on intelligence) (Savage et al., 2018). Taken together, these data suggest that reduction of OGA levels in itself could be a potential mechanism that underpins OGT-CDG (Pravata et al., 2020b). However, unlike other characterised OGT-CDG mESC cell lines, the OGT^C921Y^ variant did not induce OGA downregulation in mESCs. Furthermore, based on western blot analysis, HCF1 proteolytic processing and localisation was not affected in cultured mESCs in our study, even though *O*-GlcNAcylation and HCF1 proteolytic processing occur within the same active site of OGT. A previously reported OGT-CDG mutation affecting the TPR domain results in a similar phenotype: unaffected HCF1 processing and reduced glycosyltransferase activity (Gundogdu et al., 2018; Vaidyanathan et al., 2017). These data point to loss of *O*-GlcNAcylation on specific proteins as a likely link between OGT-CDG missense mutations and patient phenotypes.

To determine whether OGT-CDG might arise due to decreased *O*-GlcNAcylation in stem cells, we assayed self-renewal and differentiation in mESCs harbouring the C921Y mutation in *Ogt*. In these cells, the pluripotency marker ALP and the key pluripotency transcription factors Oct4 and Sox2 were significantly decreased in the presence of LIF, compared to WT colonies, implying a defect in the maintenance of an undifferentiated state. The colonies formed by OGT^C921Y^ mESCs were flat and spread out, resembling differentiating colonies in their morphology. In addition, global *O*-GlcNAcylation in OGT^C921Y^ mESC colonies was significantly reduced compared to that in OGT^WT^ mESC colonies. This is in line with a previous study indicating that preventing *O*-GlcNAcylation blocks mESC self-renewal (Jang et al., 2012). *O*-GlcNAcylation has also been shown to decrease during development (Liu et al., 2012), further supporting the hypothesis that decreased *O*-GlcNAcylation might be pathogenic through reducing stem cell self-renewal. Defects in the maintenance of pluripotency and accelerated differentiation, in particular, neuronal differentiation, have previously been associated with ID-related genes (Adamo et al., 2011; Bustos et al., 2018; Chong et al., 2016; Monderer-Rothkoff et al., 2021). However, the OGT^C921Y^ variant does not result in preferential differentiation towards a specific germ layer, despite previous research indicating that *O*-GlcNAcylation of SOX2 can promote differentiation towards an ectodermal lineage (Kim et al., 2021). These findings suggest that a potential contributor to OGT-CDG clinical manifestation is a misregulation of exit from a stem cell state. This might occur at several stages of development, with recent evidence suggesting that normal *O*-GlcNAcylation is required for maintenance not only of ESCs (Jang et al., 2012), but also of neural stem cells (White et al., 2020; Shen et al., 2021).

## MATERIALS AND METHODS

### Sequencing of patient genetic material

This study was approved by the the ethics committee of Region Zealand, Denmark (number SJ-91) and complies with the principles laid out in the Declaration of Helsinki. Parents or legal guardians gave informed consent as all patients had cognitive impairment. Consent was obtained to publish identifying photos. Whole-exome sequencing was performed on patient III:2. Subsequent screening for the OGT variant was done by Sanger sequencing in the mother, the unaffected brother (III:1) and the affected twins (III:3 and III:4). All genetic analysis was done at an accredited clinical laboratory at Amplexa Genetics A/S, Sverigesgade 24, Odense C, Denmark.

### Protein expression and purification

Human OGT^WT^ and OGT^C921Y^ constructs (full-length construct fused with an N-terminal His tag, and a 323-1044 aa shortened construct with a truncated TPR domain fused with a GST tag) were expressed in *E. coli* BL21 cells in plasmids carrying an ampicillin-resistance cassette. Transformed colonies that incorporated the plasmids were selected on ampicillin agar plates, expanded overnight at 37°C in a shaking incubator in 5× ampicillin LB broth as a starter culture, and then grown in desired quantities (6-12 l) at 37°C until reaching an optical density at 600 nm (OD_600_) of 0.5-0.6. Subsequently, the temperature of the shaking incubator was decreased to 18°C, cultures were induced with 100 µM IPTG and further cultured for 16 h. Following overnight culture, bacteria were pelleted for 45 min at 4°C, resuspended in base buffer [0.1 M Tris-HCl, pH 7.5, 0.15 M NaCl, 0.5 mM tris(2-carboxyethyl)phosphine (TCEP)] and lysed using a French press in the presence of 0.1 mg/ml DNase I, 0.5 mg/ml lysozyme and protease inhibitor cocktail (1 mM benzamidine, 0.2 mM PMSF and 5 mM leupeptin). The resulting lysates were then pelleted at 40,000 ***g*** at 4°C for 45 min. The supernatants were filtered with a 0.2 µm filter and the pellet was discarded. The clarified supernatants were exposed either to glutathione-sepharose 4B beads (MRC PPU Reagents and Services) for GST-tagged protein or NiNTA resin (MRC PPU Reagents and Services) for His-tagged protein for affinity purification. Bound proteins were either eluted using 50 mM glutathione (for GST-tagged protein) or 300 mM imidazole (for His-tagged protein) in base buffer and buffer exchanged into base buffer only, and the tag was cleaved off using 80 U of PreScission protease, purified in-house (Leong, 1999) per sample. Proteins were further purified using size-exclusion chromatography and stored in solution with 25% glycerol at –80°C.

### Enzyme assays

Michaelis–Menten kinetics of full-length recombinant OGT^WT^ and OGT^C921Y^ against 10 μM acceptor peptide (Ac-KENSPAVTPVSTA-NH_2_; synthesis as in Rafie et al., 2017) and 0-320 μM UDP-GlcNAc (Sigma-Aldrich, U4375-25MG) were tested in a fluorometric *in vitro* assay as described by Pathak and colleagues (Pathak et al., 2015). The reaction time was 4 h at room temperature. Experiments were repeated three times on different days, each repeat consisting of three technical replicates. Glycosyltransferase activity of recombinant OGT^WT^ and OGT^C921Y^ (323-1044 aa) against protein substrate was tested *in vitro* using TAB1 (7-420 aa) as described previously (Rafie et al., 2017). Experiments were repeated five times on different days.

### Differential scanning fluorimetry assay

To prepare assay mixtures, OGT^WT^ and OGT^C921Y^ (323-1044 aa) were diluted to 1.2 µM in base buffer containing 50 mM HEPES/NaOH pH 7.5, 150 mM NaCl and 0.5 mM TCEP, and mixed with 1:5000 SYPRO Orange Protein Gel Stain (Sigma-Aldrich). Then, 50 μl of assay mixture was dispensed per well in a white-bottomed quantitative PCR (qPCR) plate and each condition was performed in technical triplicate. The experiment was repeated three times on different days. The prepared assay plate was exposed to temperature increases from 25°C to 95°C with 1°C increments for 5 s each using a CFX Connect Real-Time PCR Detection System (Bio-Rad). SYPRO Orange (Thermo Fisher Scientific, S6650) fluorescence was detected after every temperature increase. Data were truncated using Excel and analysed in Prism (GraphPad) as described previously (Huynh and Partch, 2016).

### Cloning and CRISPR/Cas9

Generation of OGT^C921Y^ mESCs was performed as described previously (Pravata et al., 2019) with mutation-specific parameters. A repair template was generated to create the C921Y mutation. This was cloned as a BamHI-NotI fragment into a plasmid based on pGEX6P1. Paired gRNA sequences were selected and cloned as annealing oligonucleotides into pBABED-U6 and pX335-U6 plasmids. All plasmids were a kind gift from Thomas McCartney (University of Dundee, Dundee, Scotland). Silent mutations to remove the gRNA recognition sequences, in addition to a change at codon 935, were introduced by PCR of a gene block followed by restrictionless cloning into the cloned genomic region. The change at position 935 was introduced by the use of the C921Y wobble primer along with the reverse screening primer to generate a mutagenic PCR product and introduced by restrictionless cloning. The repair template was confirmed by DNA sequencing. Sequences of the listed custom-made reagents are listed in Table S1.

Clones were screened using paired screening primers. These generated a 600 bp PCR product, which was then digested with Fastdigest XceI (Thermo Fisher Scientific, FD1474). WT clones showed two fragments: – 277 bp and 323 bp. Successful incorporation of the repair template resulted in loss of restriction of the PCR product. Intact PCR products were confirmed by DNA sequencing. Following DNA confirmation of several clones, RNA was extracted, and one-step RT-PCR was carried out using Takara Primescript High fidelity RT-PCR kit with mOGT_solid_ex11_fwd and mOGT_solid_end_rev primers. The resulting PCR product was sequenced to confirm that the region without the repair template had not been damaged during repair and mRNA expression included the change.

### Tissue culture

AW2 mESC culture was performed as described previously (Pravata et al., 2019). The AW2 line is derived from E14-TG2a.IV (129/Ola) ESCs, kindly donated by the MRC Centre for Regenerative Medicine, Institute for Stem Cell Research, University of Edinburgh (Zhou et al., 2013). Cells were routinely tested for mycoplasma contamination and kept in strictly sterile conditions with no visible bacterial or fungal infection.

### Western blotting

Proteins were extracted from confluent mESCs. Plates or flasks with attached cells were washed twice with pre-warmed 1× PBS and then covered with 20 µl/cm^2^ of ice-cold lysis buffer (50 mM Tris-HCl, pH 7.4, 1 mM EDTA-NaOH, 1 mM EGTA-NaOH, 1% Triton X-100, 1 mM Na_3_VO_4_, 50 mM NaF, 0.27 M sucrose and 5 mM protease inhibitor cocktail). Next, prepared plates were frozen at –20°C overnight. Cells were then scraped off the plates, vortexed for 10 s and centrifuged for 45 min at 17,000 ***g*** at 4°C. Protein concentration in the clarified lysate was determined using the Pierce 660 nm Protein Assay Reagent (Thermo Fisher Scientific). For sample resolution and protein detection, 20 µg of protein was mixed with 4× NuPage LDS buffer (Invitrogen), boiled, loaded onto a 4-12% Bis-Tris gel (Invitrogen) and then transferred onto 0.2 µm nitrocellulose membranes (GE Healthcare). Following membrane blocking with 5% bovine serum albumin (BSA) in 1× TBS, the following primary antibodies were applied: anti-OGT (DM-17; Sigma-Aldrich, O6264; 1:5000), anti-O-GlcNAc (RL2; Novus Biologicals, NB300-524, 1:1000), anti-OGA (1:500, Sigma-Aldrich, SAB4200267), anti-Oct3/4 (C-10; Santa Cruz Biotechnology, sc-5279, 1:500), anti-Sox2 (Santa Cruz Biotechnology, sc-365823, 1:500), anti-histone 3 (Cell Signaling Technologies, 9715, 1:2000) and anti-α-tubulin (Proteintech, 11224-1-AP, 1:5000). After incubation with the corresponding LI-COR secondary antibodies (925-32213, 925-68073, 925-68072, 925-32212; 1:10,000), signals were detected using a LI-COR Odyssey scanning system and quantified using Image Studio Lite (LI-COR). Data were normalised and analysed in Prism. The numbers of repeats per experiment are detailed in the figure legends.

For HCF1 cleavage detection, nuclear fractionation was performed. Briefly, confluent cells were detached from plates using accutase, spun down and washed. Pelleted cells were then covered with ice-cold buffer A [10 mM HEPES pH 7.5, 1.5 mM MgCl_2_, 10 mM KCl, 0.5 mM dithiothreitol (DTT), 0.05% NP40 and protease inhibitor cocktail], vortexed and centrifuged at 900 ***g*** for 10 min at 4°C. The resulting supernatant was used as the cytoplasmic lysate fraction. The pelleted material was treated with ice-cold buffer B [5 mM HEPES pH 7.9, 1.5 mM MgCl_2_, 0.2 mM EDTA, 0.5 mM DTT and 26% glycerol (v/v)], homogenised on ice and centrifuged at 14,000 ***g*** for 10 min at 4°C. The resulting supernatant was used as the nuclear lysate fraction. Due to low protein concentration, proteins were precipitated from samples as described previously (Clark et al., 2013). Approximately 20 µg of protein was then loaded onto 4-12% gel as described above. The primary antibodies used were anti-HCF1 (Bethyl, A301-400A-M, 1:1000), anti-laminin B1 (ZL-5; Abcam, ab20396, 1:5000) and anti-β-actin (Proteintech, 66009-1-Ig, 1:10,000). After incubation with the corresponding LI-COR secondary antibody (925-68073, 925-32212; 1:10,000), signals were detected using a LI-COR Odyssey scanning system and quantified using Image Studio Lite. Data were normalised and analysed in Prism.

### RT-PCR

RNA was extracted from confluent cells using the RNeasy kit (QIAGEN). RNA purity was determined using Nanodrop 1000 (Thermo Fisher Scientific) and RNA concentration was measured using the Qubit RNA Broad Range kit (Thermo Fisher Scientific). RNA was transcribed into cDNA using the qScript cDNA Synthesis Kit (Quantabio). qPCR reactions were set up in 384-well plates in a total volume of 10 µl using PerfeCTa SYBR Green FastMix for iQ (Quanta) with 5 ng of cDNA and 250 nM forward and reverse primer mix. Three housekeeping genes (*Gapdh*, *Rn18s* and *Actb*) were used as reference genes and each condition was run in technical triplicate. No-template controls as well as no-reverse-transcription controls were used. Reactions were run in a Bio-Rad CFX 384 real-time detection system. Primer sequences are summarised in Table S2. Results were analysed using Bio-Rad CFX Manager and Prism. The numbers of repeats per experiment are detailed in the figure legends.

### ALP assay

For ALP assays, cells were well resuspended, passed through a cell strainer to remove clumps and doublets, and counted using a haemocytometer. Then, 100 OGT^WT^ or OGT^C921Y^ mESCs were plated into five wells of a six-well plate pre-treated with 0.1% (w/v) porcine gelatin (Sigma-Aldrich, G2500) in complete Glasgow's minimal essential medium (GMEM; Thermo Fisher Scientific, 21710082) as described previously (Pravata et al., 2019). Cells were grown in complete medium for 2 days to start forming colonies. From day 3 to day 7, the medium was changed in dedicated wells into no-LIF GMEM (Gibco) supplemented with 5% fetal bovine serum fraction V (Gibco), 1× non-essential amino acids (NEAA) (Gibco), 1× sodium pyruvate (Gibco) and 0.1 mM β-mercaptoethanol (Thermo Fisher Scientific). One well per plate remained without medium change. On day 7, the medium was aspirated, and colonies were washed twice with 1× PBS and fixed with 1% paraformaldehyde (PFA) for 2 min. Fixed colonies were stained with the Alkaline Phosphatase kit (Merck) according to the manufacturer's instructions. ALP-treated plates were scanned on a flat-bed scanner (Epson V800), and colonies were counted and scored (undifferentiated, mixed or differentiated) under a microscope (Axiovert 40 C, Zeiss). Colonies were subsequently re-stained with Coomassie Blue (Abcam, ISB1L) to visualise ALP-negative colonies, and plates were re-scanned. Experiments were repeated three times using three cell lines per genotype. Colony diameters were determined using ImageJ based on Coomassie Blue scans using a macro. Briefly, colonies were segmented by creating a binary image by setting an automatic threshold using the ‘MaxEntropy’ algorithm and measured using the ‘Analyze Particles’ function. Data were analysed using Prism.

### Cell cycle analysis

Cell cycle analysis was performed using near-confluent OGT^C921Y^ (two lines) and OGT^WT^ (one line) mESCs collected over three subsequent passages. DNA content was assessed using Abcam Propidium Iodide kit (ab139418), according to the manufacturer's instructions. Flow cytometry analysis was performed on a BD FACSCanto flow cytometer (BD Biosciences). Data were analysed using FlowJo and Prism software.

### Immunofluorescence analysis of colonies

Colonies were plated on coverslips coated with 0.1% (w/v) porcine gelatin in complete GMEM as described above. Six days after plating, colonies were fixed for 15 min using 4% PFA. PFA was then quenched with 1× TBS for 10 min. Fixed colonies were then washed with 1× PBS twice for 5 min each. Samples were subsequently permeabilised and precipitated using ice-cold 90% (w/w) methanol at −20°C for 10 min. Samples were then washed twice with 1× PBS and blocked with blocking buffer (1× PBS, 1% Tween 20 and 5% BSA) for at least 15 min. Primary antibodies [anti-Oct3/4 (D6C8T; Cell Signaling Technology, 83932, 1:1000), anti-Sox2 (D9B8N; Cell Signaling Technology, 23064, 1:1000), anti-Pax6 (H-295; Santa Cruz Biotechnology, sc-11357, 1:1000), anti-Sox17 (Santa Cruz Biotechnology, sc-130259, 1:1000) and anti-Brachyury (D10; Santa Cruz Biotechnology, sc-166962, 1:1000)] were added in blocking buffer overnight. Following primary antibody staining, cells were washed three times for 15 min with 1× PBS and secondary antibodies [donkey anti-rabbit Alexa Fluor 488 (Thermo Fisher Scientific, A21206), donkey anti-mouse Alexa Fluor 647 (Thermo Fisher Scientific, A31571), 1:2000] were added in blocking buffer. Colonies were washed with 1× PBS and DAPI, mounted and imaged on a confocal microscope (Leica SP8 with HC PL APO CS2 20×, 0.75 Dry objective). Images were analysed using an ImageJ macro. Briefly, nuclear regions were determined by performing a series of global and local thresholding steps. Non-nuclear regions were determined based on subtraction of nuclear regions from a binary image created by applying an automatic threshold to individual channels using the ‘MinError’ algorithm.

## Supplementary Material

10.1242/dmm.049132_sup1 Supplementary information
