# Supplementary material for: An O-GlcNAc transferase pathogenic variant linked to intellectual disability affects pluripotent stem cell self-renewal
Source: Dis Model Mech. 2023 Jun 19;16(6):dmm049132. doi: 10.1242/dmm.049132 (PMC10309585; doi:10.1242/dmm.049132)
Supplement: Supplementary information [file dmm-16-049132-s1.pdf]

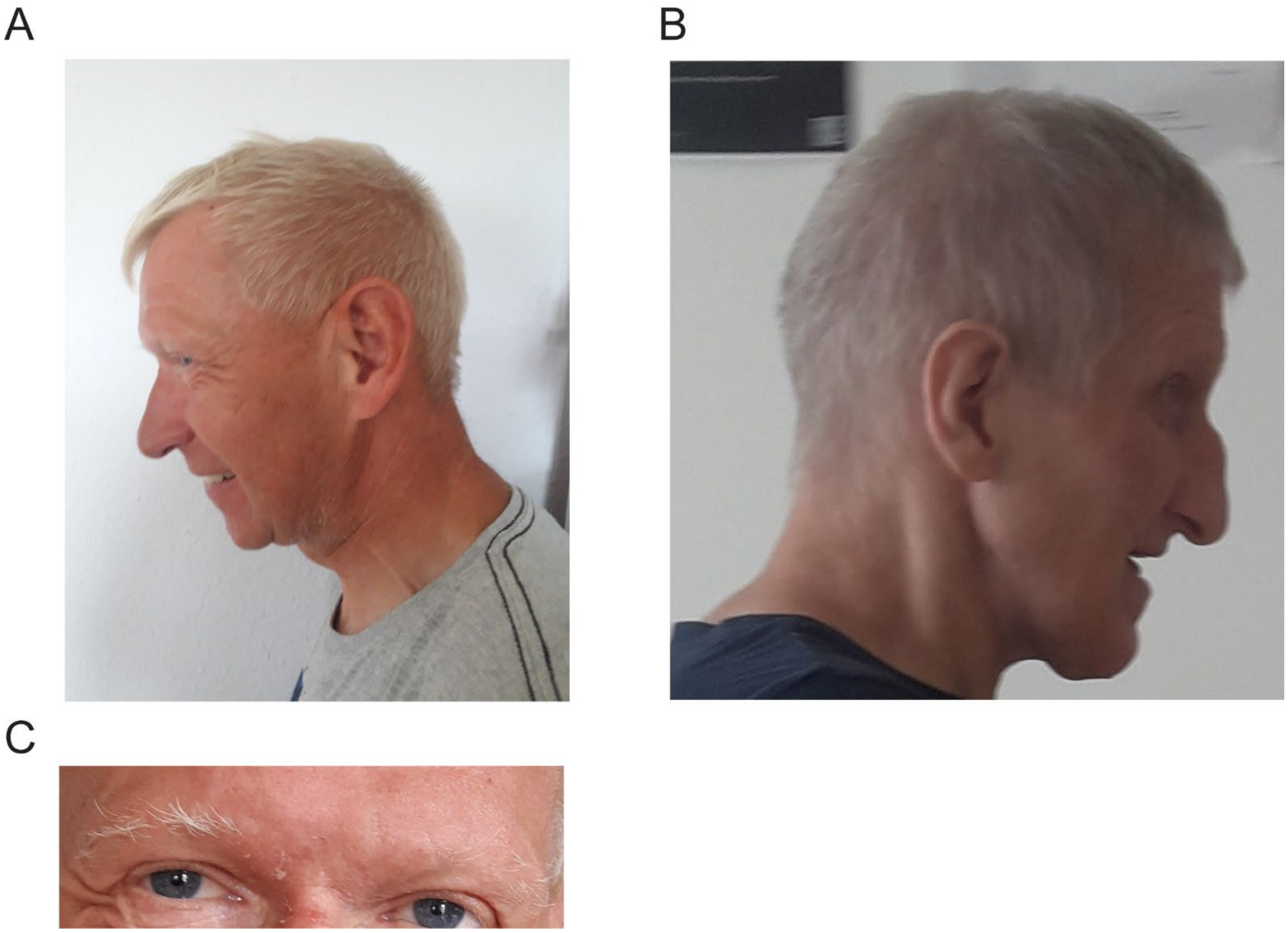

Fig. S1. Profile photos and close up view of patient's eyebrows.

a) and b) Profile photographs of affected brothers carrying OGT^{C921Y} variant.

c) Proband III.2 has sparse eyebrows.

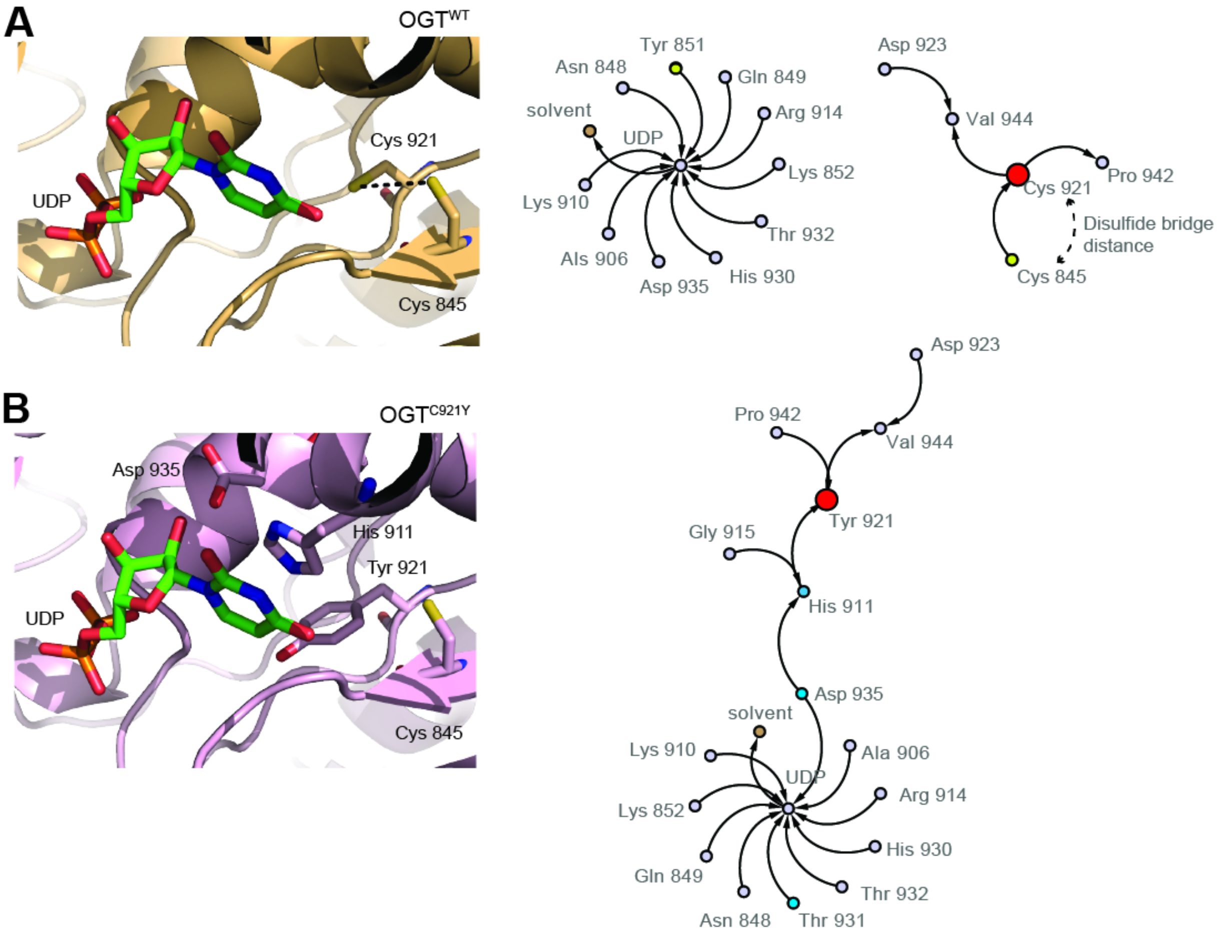

Fig. S2. Model of the OGT C921Y substitution and changes in interactions in the catalytic core.

a) The PDB 5LWV containing a UDP molecule sitting in the active site (green sticks) and a fusion protein peptide, removed for our analysis, was used a template for the OGT^{WT} protein. Averaged MD trajectory calculated after 1000 poses is shown in the left side. For clarity, cysteine 921 is represented as a red circle.

b) The OGT^{C921Y} variant was modelled over the PDB 5LWV with the same modifications as described for OGT^{WT}. Averaged MD trajectory calculated after 1000 poses is shown in the left side. For clarity the mutant tyrosine 921 is represented with a red circle. All key residues are labelled.

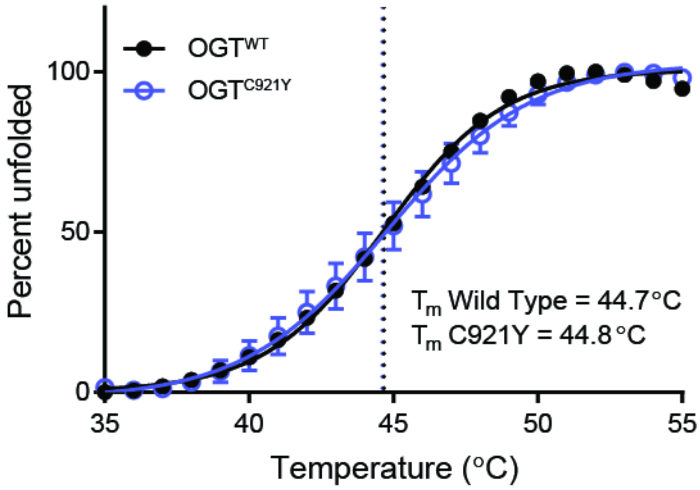

Fig. S3. Melting temperature determination for OGT^{WT} and OGT^{C921Y} (323 – 1044).

Thermal shift assay performed with recombinant OGT^{WT} and OGT^{C921Y} hOGT (323-1041). This experiment was performed three times, using three technical replicates each time. Error bars represent standard deviation.

Disease Models & Mechanisms • Supplementary information

A

```
human   860  AIVYCNFNQLYKIDPSTLQMWANILKRVPNSVLWLLRFPAVGEPNIQQYAQNMGLPQNRI     900
mouse        AIVYCNFNQLYKIDPSTLQMWANILKRVPNSVLWLLRFPAVGEPNIQQYAQNMGLPQNRI     900
             ************************************************************

human        IFSPVAPKEEHVRRGQLADVCLDTPLCNGHTTGMDVLWAGTPMVTMPGETLASRVAASQL     960
mouse        IFSPVAPKEEHVRRGQLADVCLDTPLCNGHTTGMDVLWAGTPMVTMPGETLASRVAASQL     960
             ************************************************************

human        TCLGCLELIAKNRQEYEDIAVKLGTDLEYLKKVRGKVWKQRISSPLFNTKQYTMELERLY     1020
mouse        TCLGCLELIAKSRQEYEDIAVKLGTDLEYLKKIRGKVWKQRISSPLFNTKQYTMELERLY     1020
             ***********.********************:***************************

human        LQMWEHYAAGNKPDHMIKPVEVTESA*     1046
mouse        LQMWEHYAAGNKPDHMIKPVEVTESA*     1046
             **************************
```

B

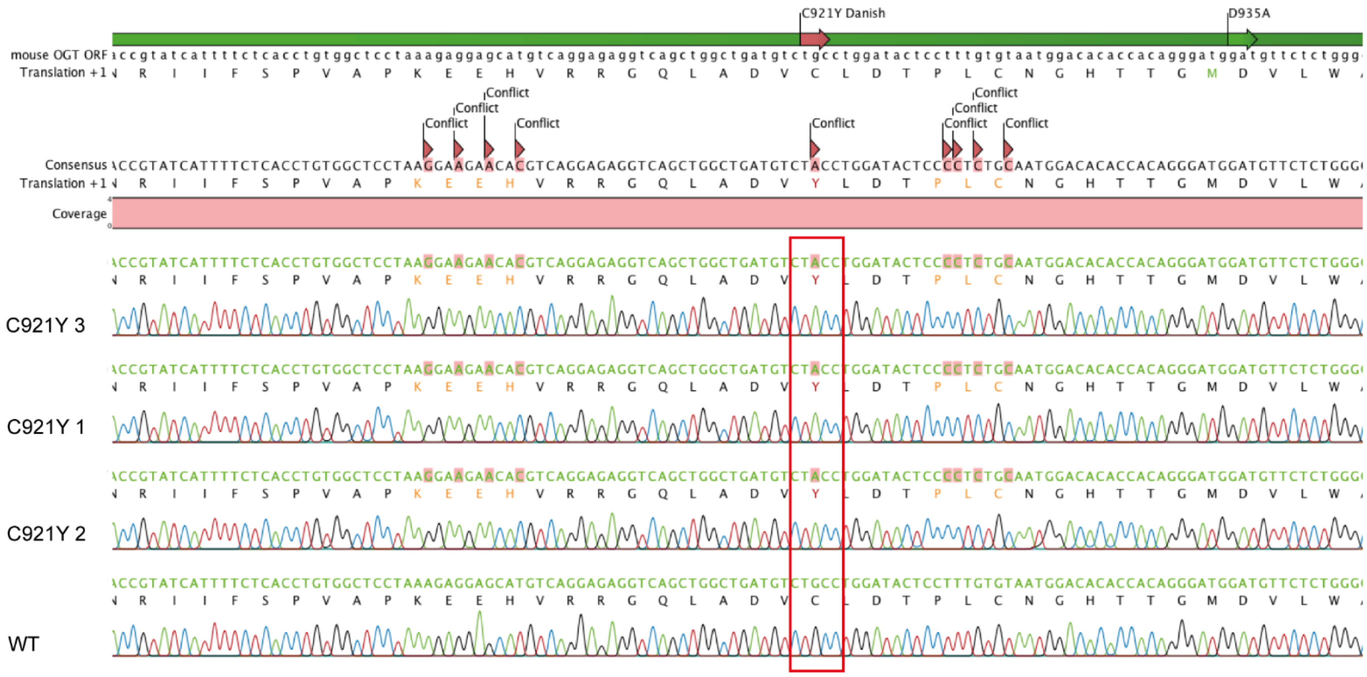

Fig. S4. Generation of OGT^{C921Y} mESC lines.

a) Sequence alignment of human and mouse OGT (amino acids 840 – 1046). Cysteine 921 is highlighted in red.

b) DNA sequencing of generated OGTC921Y mESC clones (chromas 1 – 3) and wild type mESC (Chroma 4). Positions corresponding to cysteine 921 are highlighted in red rectangle.

Disease Models & Mechanisms • Supplementary information

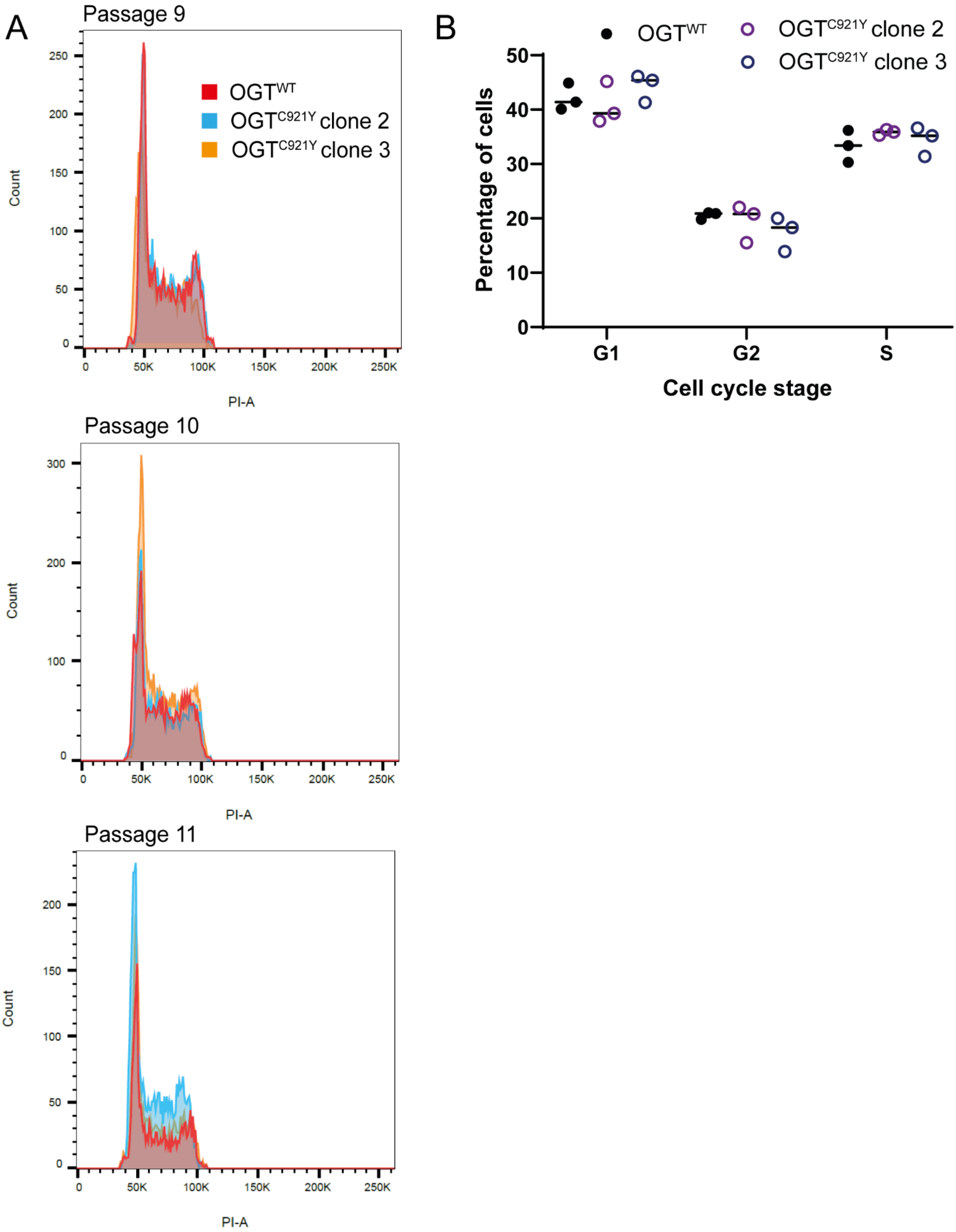

Fig. S5. Cell cycle analysis of OGT^{WT} and OGT^{C921Y} mESC.

a) Cell cycle profiles of OGT^{WT} and OGT^{C921Y} across three passages using two mutant and one wild type cell line. We aimed to perform cell cycle profiling with the earliest available passages, which is the reason for not using all three cell OGT^{C921Y} cell lines in this experiment.
b) Plot of percentages of cells in each cell cycle stage.

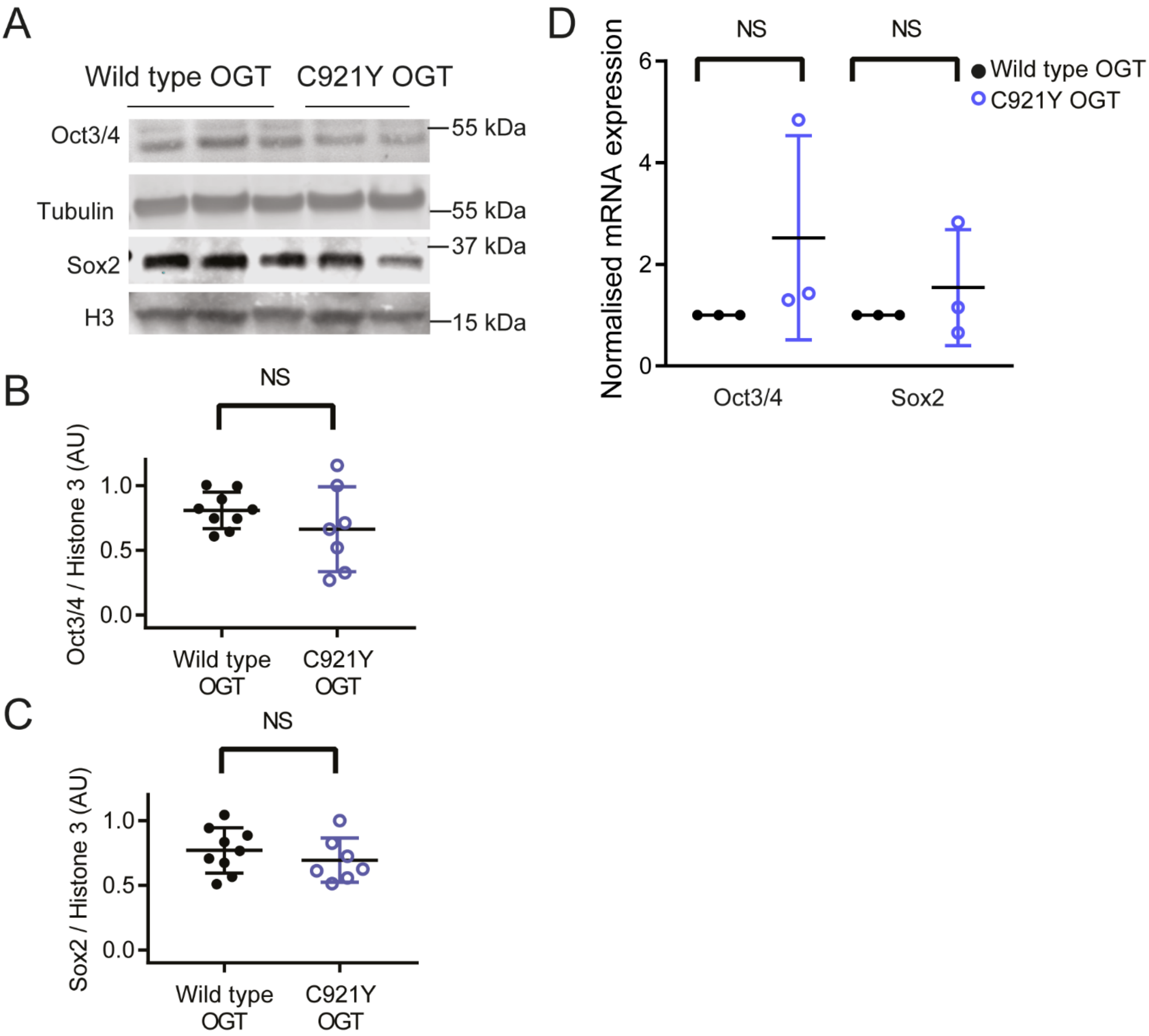

Fig. S6. Oct3/4 and Sox2 expression in OGT^{WT} and OGT^{C921Y} mESC propagated in LIF.

The quantification shown in panels B–D is based on results obtained from two to three different cell clones per genotype and repeated over three passages per clone.

a) Immunoblot of Oct3/4, Sox2, Tubulin and Histone3.

b) Quantification of Oct3/4 signal relative to tubulin. Unpaired *t* test, *p* value = 0.251. Error bars represent standard deviation.

c) Quantification of Sox2 signal relative to Histone 3. Unpaired *t* test, *p* value = 0.401. Error bars represent standard deviation.

d) mRNA expression of Oct3/4 and Sox2. Unpaired *t* test, Sox *p* value = 0.455, Oct3/4 *p* value = 0.260. Each data point represents normalised mean expression calculated from three separate RT-PCR runs. Each RT - PCR run was set up using several OGTWT and OGTC921Y as biological replicates. Error bars represent standard deviation.

Disease Models & Mechanisms • Supplementary information

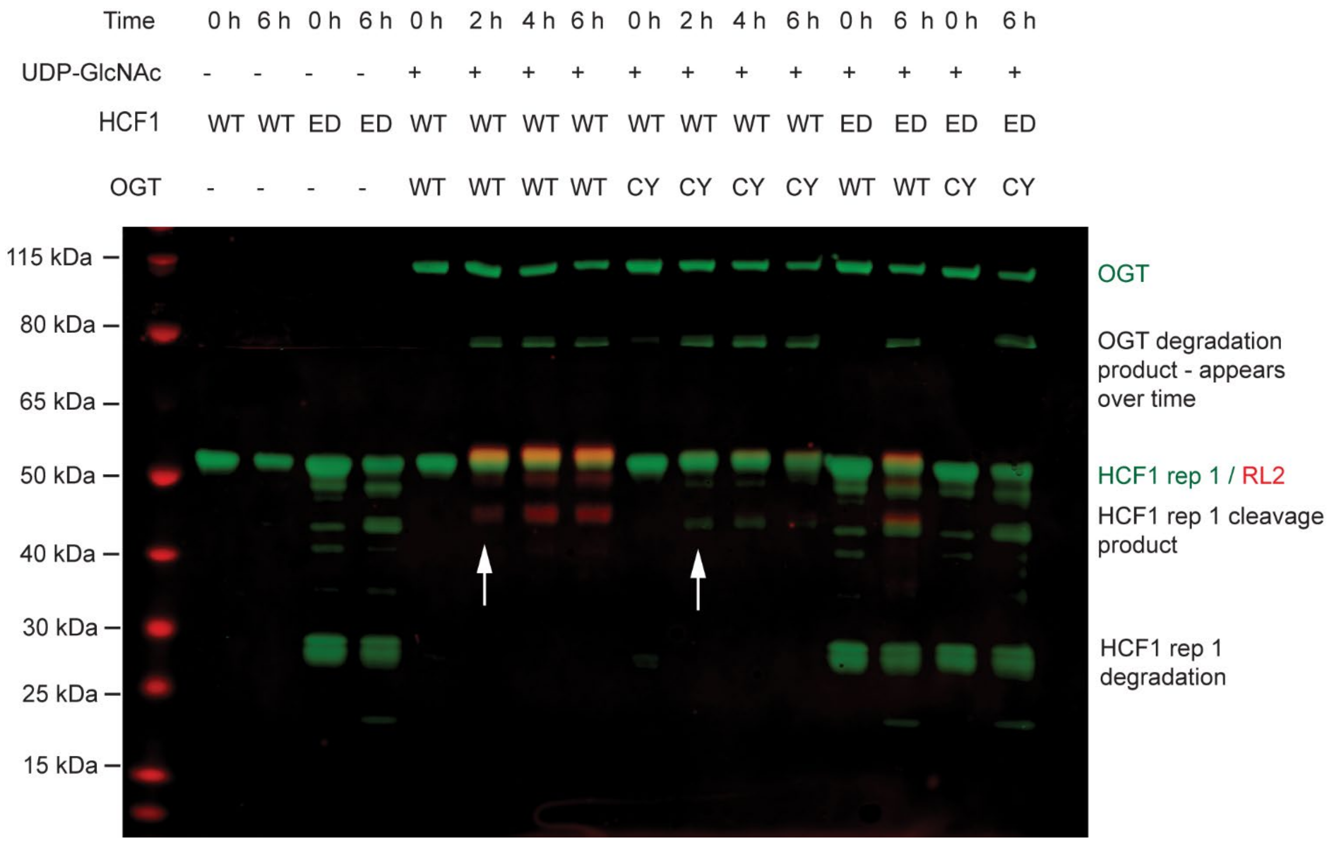

Fig. S7. HCF1 processing by recombinant OGT^{WT} and OGT^{C921Y}.

Immunoblot of HCF1 proteolytic fragments produced in a time course *in vitro* reaction using the following human recombinant proteins: wild type (WT) and uncleavable (ED) HCF1 repeat 1 (HCF1rep1); OGT^{WT} and OGT^{C921Y}. HCF1 and OGT were detected in the green channel, O-GlcNAc (RL2) was detected in the red channel.

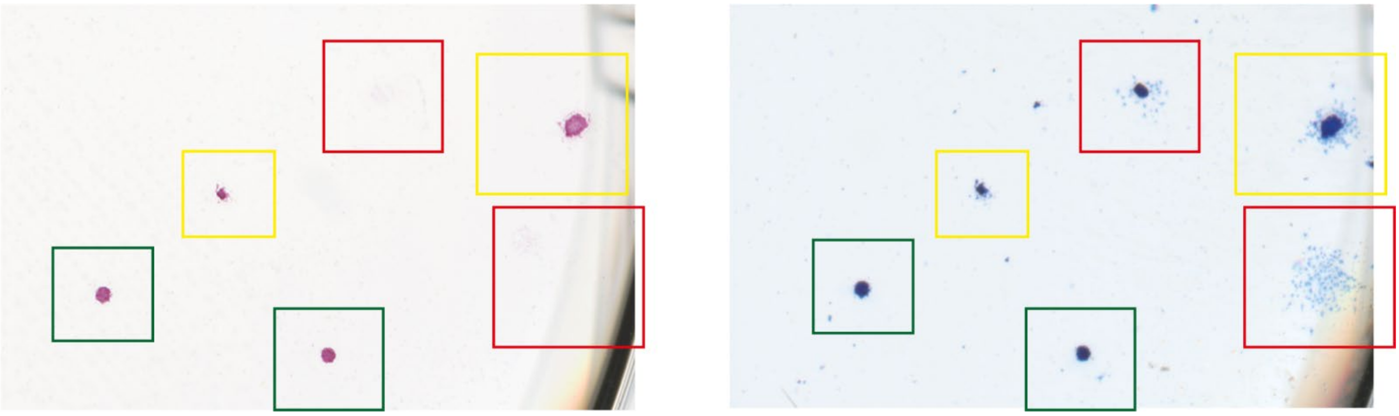

Fig. S8. Colony scoring criteria. Images corresponding to the same section of a 6-well plate are shown. Image in the left represents colonies stained with ALP and the image on the right represents colonies stained with Coomassie. Compact colonies with strong ALP staining and no signs of differentiation were scored as undifferentiated (green rectangle), ALP positive colonies with signs of differentiation around the perimeter were scored as mixed (yellow rectangle) and dispersed colonies with no ALP signal were scored as differentiated (red rectangle).

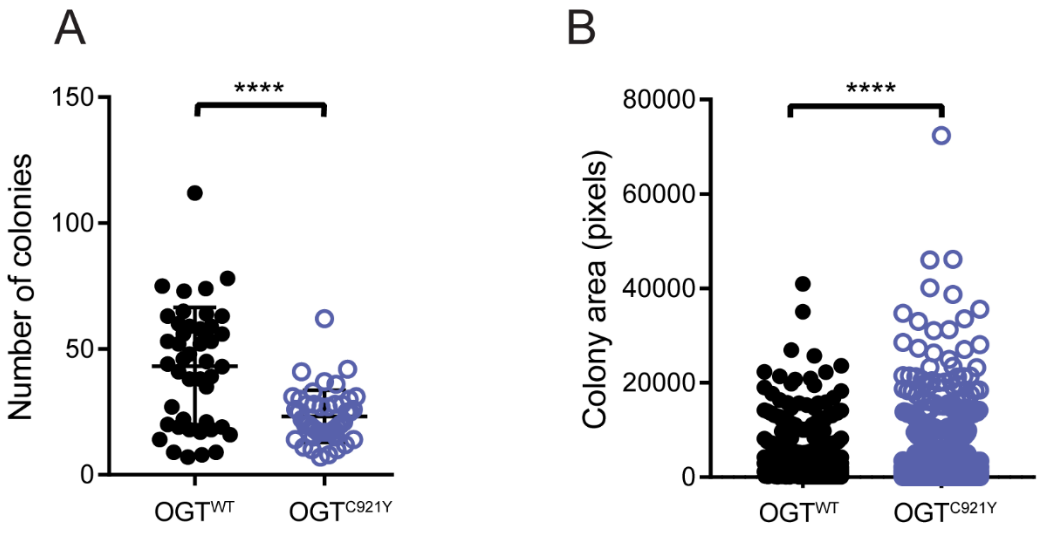

Fig. S9. Number and size of OGT^{WT} and OGT^{C921Y} colonies.

(a) Total number of colonies produced by wild type or OGT^{C921Y} mESCs in any condition. Unpaired t test, n = 45 wells scored, $p < 0.0001$. Error bars represent standard deviation.

(b) Mean colony area with SEM measured by automated ImageJ macro based on scans of colonies stained with Coomassie. Unpaired t test, wild type n = 2270 measured colonies, OGT^{C921Y} n = 1533 measured colonies, $p < 0.0001$.

Disease Models & Mechanisms • Supplementary information

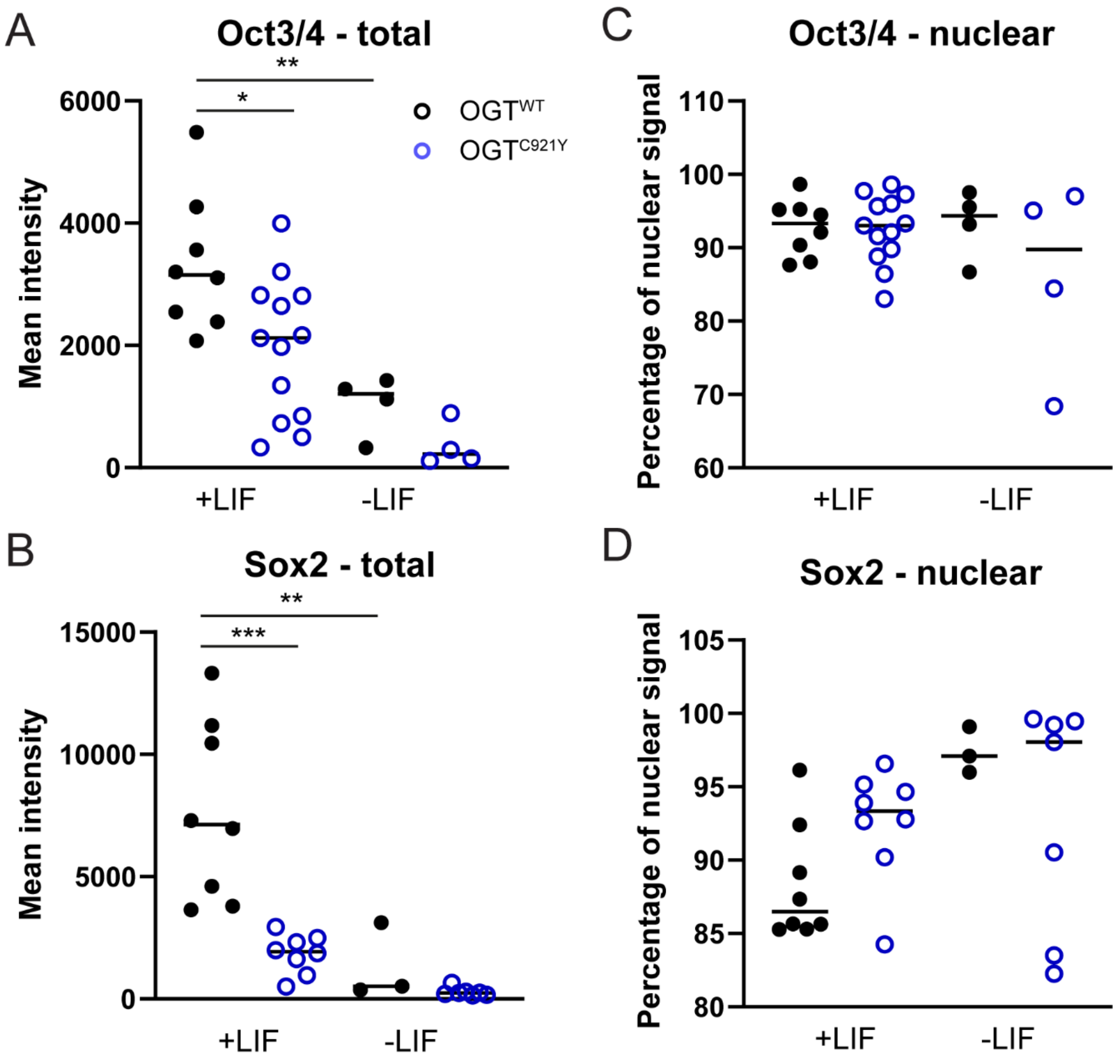

Fig. S10. Oct3/4 and Sox2 levels in OGT^{WT} and OGT^{C921Y} colonies.

(A, B) Each data point represents the mean intensity per colony calculated as sum signal intensity divided by colony area. Oct3/4 +LIF OGT^{WT} versus OGT^{C921Y}, *p* value = 0.035 (One-way ANOVA followed by Sidak's multiple comparison test). Oct3/4 +LIF OGT^{WT} versus -LIF OGT^{C921Y}, *p* value = 0.006 (One-way ANOVA followed by Sidak's multiple comparison test). Sox2 +LIF OGT^{WT} versus OGT^{C921Y}, *p* value = 0.0001 (One- way ANOVA followed by Sidak's multiple comparison test). Sox2 +LIF OGT^{WT} versus -LIF OGT^{WT}, *p* value = 0.002 (One-way ANOVA followed by Sidak's multiple comparison test). (D – F) Each data point represents the percentage of nuclear signal calculated as sum nuclear signal divided by sum signal intensity. Two lines per genotype over two passages were used for this experiment. All the colonies present on a coverslip were imaged. One-way ANOVA showed no significant differences in nuclear signal between tested groups.

Disease Models & Mechanisms • Supplementary information

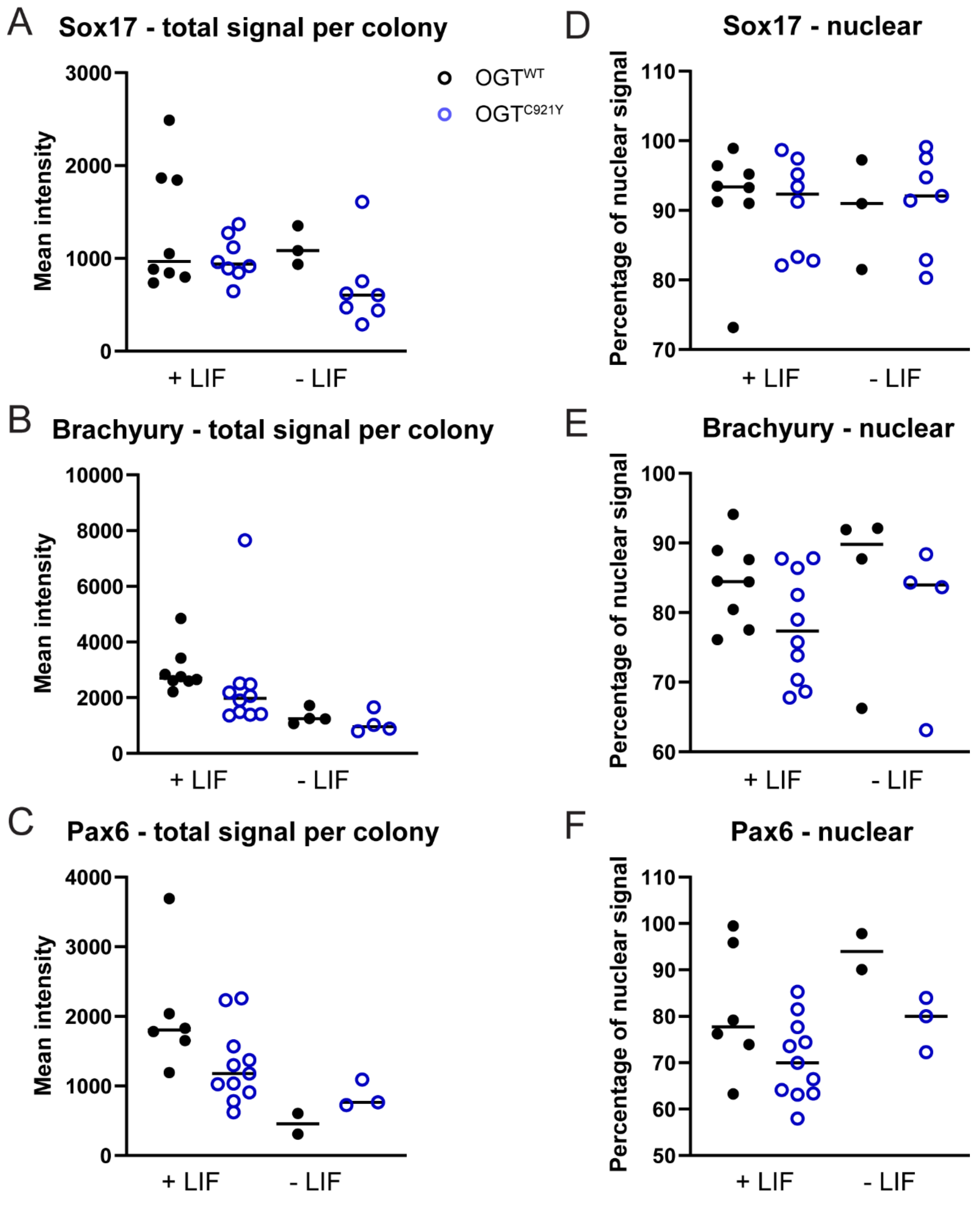

Fig. S11. Sox17, Brachyury and Pax6 levels in OGT^{WT} and OGT^{C921Y} colonies.

(A – C) Each data point represents the mean intensity per colony calculated as sum signal intensity divided by colony area. (D – F) Each data point represents the percentage of nuclear signal calculated as sum nuclear signal divided by sum signal intensity. Two lines per genotype over two passages were used for this experiment. All the colonies present on a coverslip were imaged. One-way ANOVA showed no significant differences between tested groups.

Disease Models & Mechanisms • Supplementary information

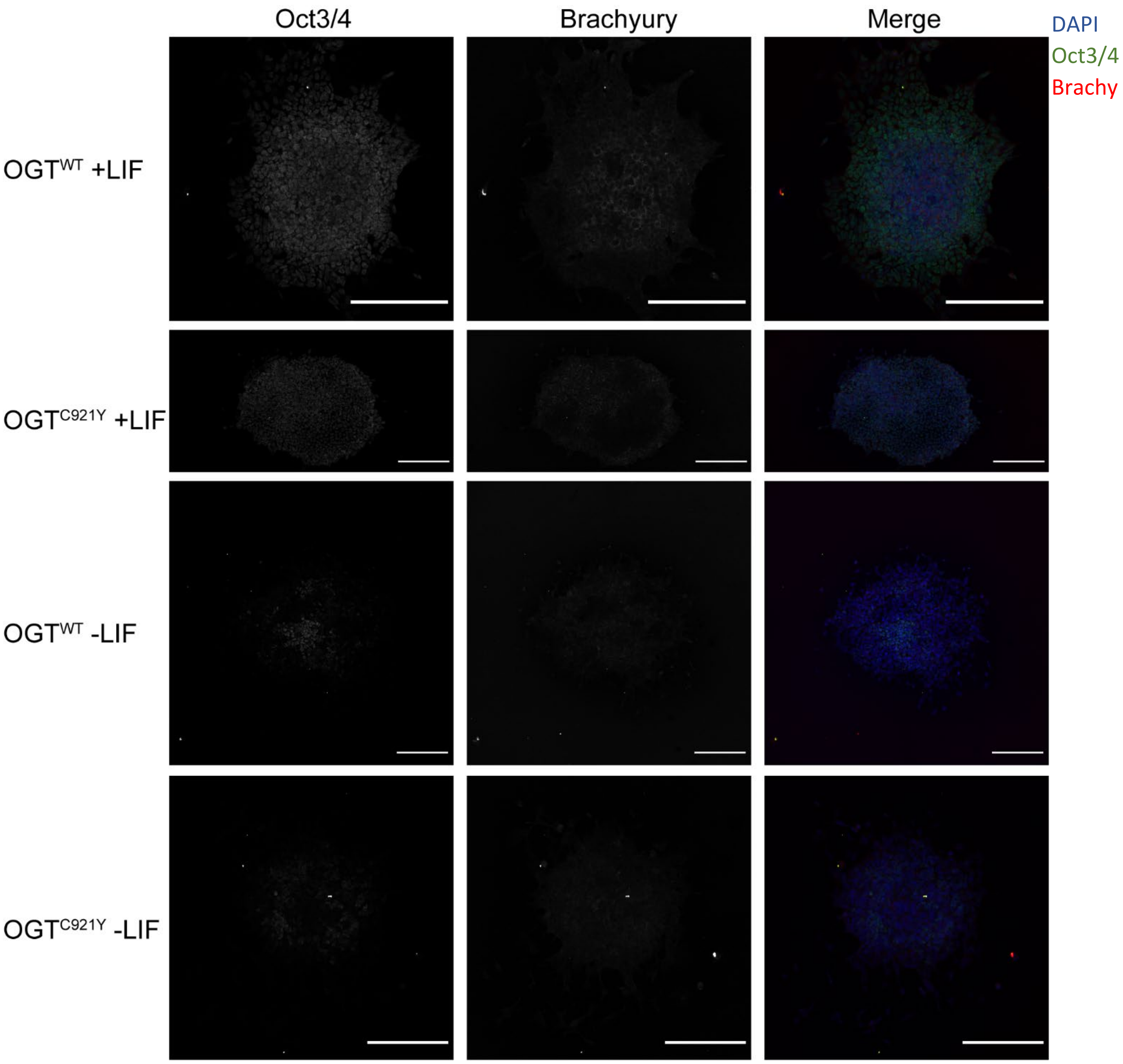

Fig. S12. Representative images of Oct3/4 and Brachyury staining in OGT^{WT} and OGT^{C921Y} colonies. Scale bar = 200 μM.

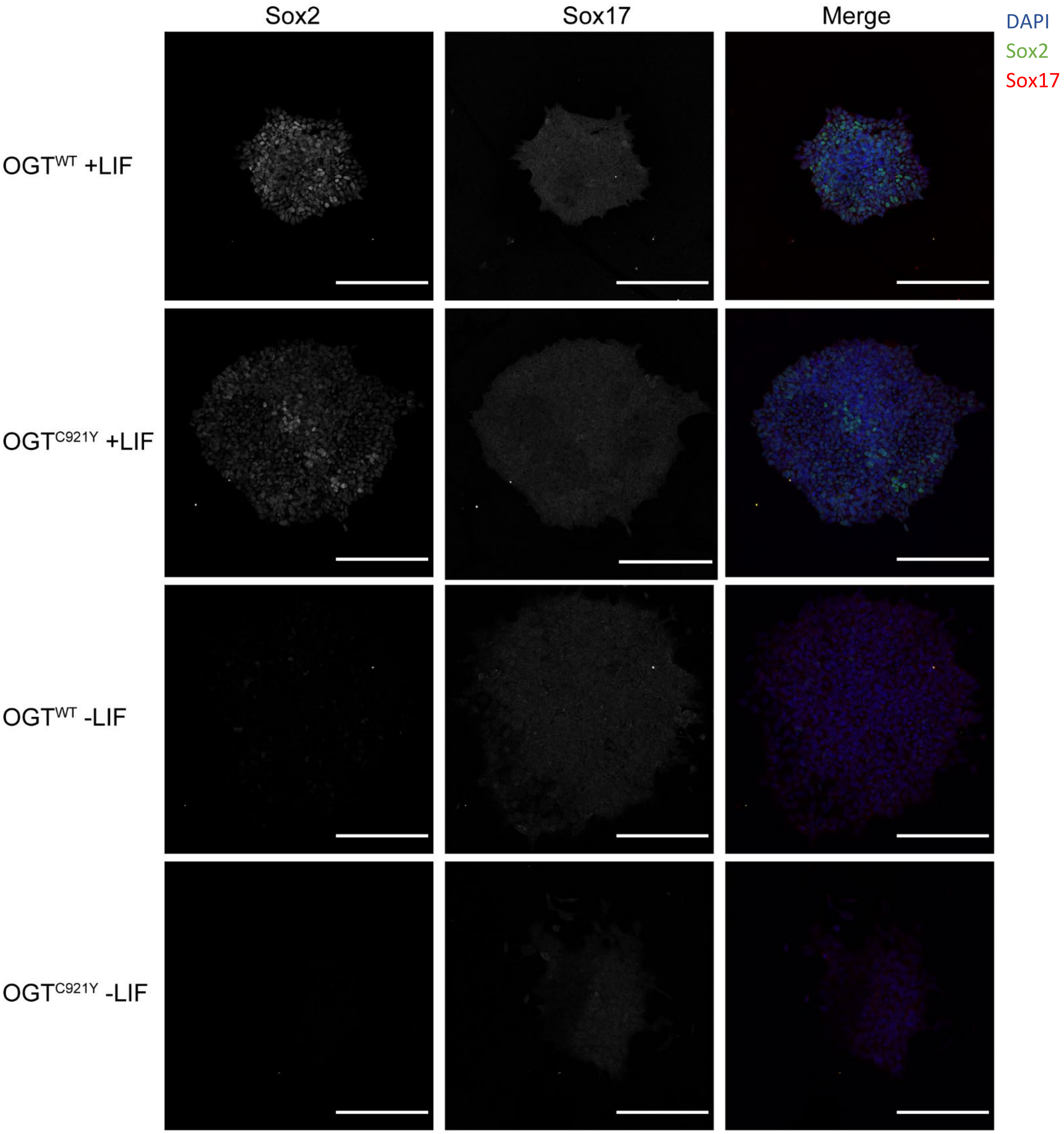

Fig. S13. Representative images of Sox2 and Sox17 staining in OGT^{WT} and OGT^{C921Y} colonies. Scale bar = 200 μM.

Disease Models & Mechanisms • Supplementary information

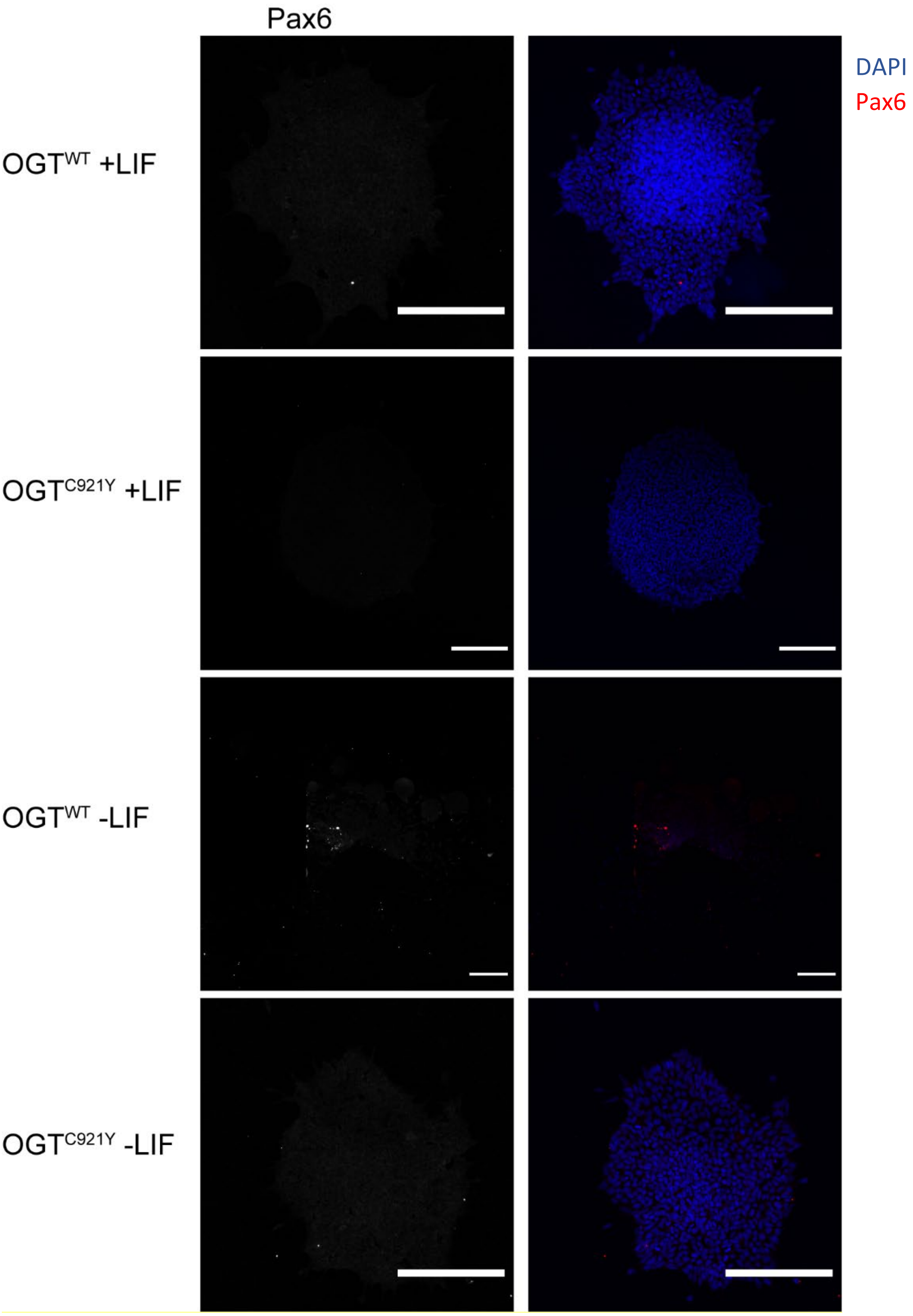

Fig. S14. Representative images of Pax6 staining in OGT^{WT} and OGT^{C921Y} colonies.
Scale bar = 200 μM.

Disease Models & Mechanisms • Supplementary information

Table S1. Sequences of custom-made reagents used for generation of OGT^{C921Y} mESC lines.

Guide RNA	M935_left_F	CACC G ctcctgacatgctcctcttt
Guide RNA	M935_left_R	AAAC aaagaggagcatgtcaggagC
Guide RNA	M935_right_F	CACC G ctggatactcctttgtgtaa
Guide RNA	M935_right_R	AAAC ttacacaaaggagtatccag C
Genomic DNA	M935V6_fwd	aaaGGATCC acacaaaaacaacaactatatacatgaagg
Genomic DNA	M935V6_rev	aaaGCGGCCGC tttctctgtgtaataaagcccttaaatatc
Sequencing	M935seq	Ttggcaagctctgccaatag
Geneblock	M935 Wobble	ggagaacccaatattcaacaatatgcacaaaatatgggccttccccagaaccgt atcattttctcacctgtggctcctaaGgaAgaAcaCgtcaggagaggtcagctg gctgatgtctgcctggatactccCCtCtgCaatggacacaccacagggatgg CCgttctctgggcaggaacacccatggtgactatgccagg
Mutagenesis	C921Y_wobble_F	GAGGTCAGCTGGCTGATGTCTaCCTGGATACTCCCCTC TGCAATGGACACACCACAGGGATGGatGTTCTCTGGGC AGGAACACC
Genotyping	M935_screen_F	atgtggttttagggactttgtgagctc
Genotyping	M935_screen_R	gagaggatggtgccaagtattcaggc
RT-PCR	mOGT_solid_ex11_fwd	ggaatatcccagaagcaatagcttcttac
RT-PCR	mOGT_solid_end_rev	ggctgactcggtgacttcaacaggc
RT-PCR - sequencing	mOGT_ex18_seqF	tcctgatggaggtgacaatc

Table S2. Sequences of qPCR primers.

**Primer**	**Sequence (5’ -> 3’)**
Actb Forward	AGATCAAGATCATTGCTCCTCCT
Actb Reverse	ACGCAGCTCAGTAACAGTCC
Gapdh Forward	GGAGAGTGTTTCCTCGTCCC
Gapdh Reverse	*ACTGTGCCGTTGAATTTGCC*
18S Forward	*CTCAACACGGGAAACCTCAC*
18S Reverse	*CGCTCCACCAACTAAGAACG*
Ogt Forward	*CCCCCTGAGCCCTTCAAAAC*
Ogt Reverse	*TCGTTGGTTCTGTACTGTCGG*
Oga Forward	*TGCAGTGGTTAGGGTGTCG*
Oga Reverse	*AGCAAACGCTGGAACTCTCC*
HCFC1 Forward	*GTGACCCAGGTGGTGCTAAA*
HCFC1 Reverse	*TTGACAGCAGAGACGGTGAC*
Oct4 Forward	*GGTGGAACCAACTCCCGAGG*
Oct4 Reverse	*ACCTTTCCAAAGAGAACGCCC*

Disease Models & Mechanisms • Supplementary information
